# Ultra‐Tough Hybrid Supramolecular Gelatin Hydrogels

**DOI:** 10.1002/smll.75441

**Published:** 2026-09-02

**Authors:** Patrick Shakari, Christos Leliopoulos, Hamidreza Mokhtari, Rohith Pavan Parvathaneni, Afroditi Kontse, Jöns Hilborn, Oommen P. Varghese

**Affiliations:** ^1^ Program of Macromolecular Chemistry Department of Chemistry – Ångström Laboratory Uppsala University Uppsala Sweden; ^2^ Translational Chemical Biology Group Science For Life Laboratory Division of Macromolecular Chemistry Department of Chemistry‐Ångstrom Laboratory Uppsala University Uppsala Sweden

**Keywords:** coacervation, gelatin, regenerative engineering, supramolecular, tannic acid, tough hydrogels

## Abstract

Natural load‐bearing tissues derive mechanical performance from sequential self‐assembly, in which collagen fibrils first compact and are then stabilized by enzymatic covalent crosslinking. Replicating this design in synthetic hydrogels while limiting swelling and preserving toughness and viscoelasticity remains challenging. Here, we report a gelatin‐based hybrid hydrogel in which tannic acid‐mediated coacervation creates a compact acidic microenvironment that triggers in situ covalent crosslinking. Tannic acid induces rapid phase separation, lowers the local pH to ∼2.7, and promotes proton‐catalyzed ring opening of the preloaded bis‐oxazoline crosslinker, resulting in diester‐linked covalent stabilization of the compacted gelatin network. This decouples physical compaction from covalent stabilization and avoids the metastability of supramolecular tannic acid‐gelatin networks. The optimized formulation showed a ∼60‐fold increase in storage modulus (G′ ∼54 kPa) relative to acid‐swollen controls and combined high extensibility (elongation at break ∼900%), tensile strength (∼2.0 MPa), and toughness (5.46 MJ m^−3^). It also retained cyclic dissipation (4.21 MJ m^−3^ in the first cycle) and long‐term structural stability in water. In vitro, the hydrogels supported adhesion, spreading, and proliferation of human dermal fibroblasts over 3 days. Together, these results show that tannic acid‐mediated compaction coupled with acid‐triggered covalent crosslinking provides an effective route to strong, extensible, and dissipative gelatin hydrogels.

## Graphical abstract

Tannic acid‐mediated coacervation compacts gelatin into a dense supramolecular network while generating an acidic microenvironment that activates pre‐embedded bis‐oxazoline crosslinkers. The resulting hybrid hydrogel combines covalent stabilization with reversible energy dissipation, producing high strength, extreme extensibility, substantial toughness, and long‐term aqueous stability while maintaining early fibroblast cytocompatibility.

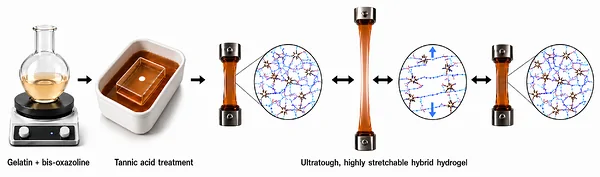

## 1 Introduction

Load‐bearing biological tissues, such as cartilage, tendons, ligaments, and skin, exhibit extraordinary mechanical performance, combining high stiffness, extreme toughness, and remarkable extensibility [1, 2]. These properties arise from the hierarchical assembly of the collagen‐rich extracellular matrix, in which secreted tropocollagen molecules self‐assemble into fibrils that are subsequently organized and compacted through cell‐generated forces and osmotic effects. Long‐term stabilization is then achieved through enzymatic crosslinking, for example via lysyl oxidase [2, 3, 4, 5]. Crosslinking synergizes with deformation mechanisms such as fibril sliding, while hydration modulates intermolecular interactions and molecular mobility, collectively shaping the nonlinear mechanical response of the tissue [2, 6, 7].

Historically, human ingenuity has mimicked aspects of this process to obtain tough materials, such as leather, by tanning collagen‐rich hides with plant‐derived polyphenols such as tannic acid. These polyphenols form multipoint hydrogen bonds and hydrophobic interactions with collagen, resulting in self‐assembly to obtain tougher materials [8, 9, 10]. In such systems, the macroscopic geometry largely corresponds to the flat anatomical shape of the animal hide, which is then stabilized during tanning through extensive supramolecular interactions. This stabilization constrains subsequent molding, injection, or additive manufacturing into complex three‐dimensional geometries required for mechanically demanding biomedical scaffolds such as tendon, ligament, or cartilage implants.

To overcome this limitation, gelatin, the thermally denatured derivative of collagen, offers a solution‐processable biomaterial platform [11, 12, 13, 14, 15]. Gelatin retains native cell‐binding motifs, remains susceptible to enzymatic degradation, and partially renatures into triple helices upon cooling to form a physically crosslinked network [11, 12, 13, 14, 15]. Despite these advantages, gelatin hydrogels remain mechanically weak, typically exhibiting storage moduli in the low‐kPa range and low work of extension (≈1–100 kJ m^−3^), which largely confines their use to non‐load‐bearing applications. By contrast, recent advances in tough hydrogels have yielded materials with energy densities on the order of 1–10 MJ m^−3^ through extreme extensibility and sacrificial‐bond dissipation [16, 17, 18, 19, 20]. However, these systems are predominantly based on non‐degradable synthetic polymer networks and are therefore poorly suited for in vivo tissue regeneration and repair. Bridging this performance gap within biodegradable, protein‐based systems remains an unmet challenge.

Efforts to address this limitation have focused on reinforcing gelatin through supramolecular interactions, including catechol‐ and polyphenol‐mediated bonding, as well as hybrid‐network designs that introduce dissipative mechanisms [21, 22, 23, 24]. Among these, tannic acid, a hydrolyzable gallotannin composed of polygalloyl‐β‐D‐glucose, has emerged as a versatile reinforcement agent [21, 22]. Tannic acid forms multivalent, reversible interactions with gelatin chains, enabling network compaction and stiffness enhancement while preserving bioactivity [23, 25, 26, 27, 28]. Yet, tannic acid‐gelatin systems that rely predominantly on supramolecular interactions remain intrinsically unstable. Increasing tannic acid content typically yields dense but brittle networks with reduced extensibility, while exposure to aqueous environments leads to swelling, tannic acid leaching, and progressive mechanical degradation [23, 24, 29]. As a result, purely physical tannic acid‐gelatin networks are unable to maintain structural integrity under physiological conditions [23, 24].

Here, we introduce a strategy that exploits the intrinsic acidification generated during tannic acid‐mediated coacervation as an internal trigger for covalent crosslinking. Incorporating a bis‐oxazoline crosslinker into the gelatin network before tannic acid exposure enables tannic‐acid‐driven compaction to occur together with proton‐catalyzed, bis‐oxazoline‐mediated covalent stabilization. This decoupling of network formation and stabilization enables shapeable precursors that can be permanently crosslinked without disrupting the compact architecture. In doing so, the approach integrates supramolecular energy dissipation with covalent network integrity within a single biodegradable material platform. The resulting hydrogels combine high strength, extensibility, and viscoelastic dissipation with long‐term stability in aqueous environments, thereby establishing a route to mechanically robust, biodegradable materials that help bridge the gap between conventional protein hydrogels and load‐bearing biological tissues.

## 2 Results and Discussion

### 2.1 Tannic Acid‐Driven Coacervation Rapidly Compacts Gelatin and Strengthens the Network Over Time

The first key challenge in developing tough materials using gelatin is to identify strategies that induce self‐assembly and network organization similar to collagen in native tissue. For this purpose, we explored the role of tannic acid, a well‐known polyphenol that can both dehydrate the samples and facilitate hydrophobic interactions and hydrogen bonding [21, 22, 30]. Upon immersion of the physically crosslinked gelatin hydrogel (Gel‐PHy) in tannic acid solutions (5–40 wt.%), the network underwent rapid supramolecular compaction into a dense structure, hereafter termed the tannic‐acid‐treated gelatin physical hydrogel (Tan‐GelPHy) (Figure 1A). This transition is driven by the interplay between electrostatic effects and multivalent supramolecular complexation. In contrast to conventional acidic conditions, which typically induce osmotic swelling in gelatin networks through protonation of the backbone and the resulting Donnan osmotic pressure [31, 32], tannic acid treatment suppresses this response. Instead, multivalent reversible interactions between tannic acid and gelatin chains bridge and constrain the network, preventing expansion despite the low‐pH environment [21, 22, 30].

**FIGURE 1:**
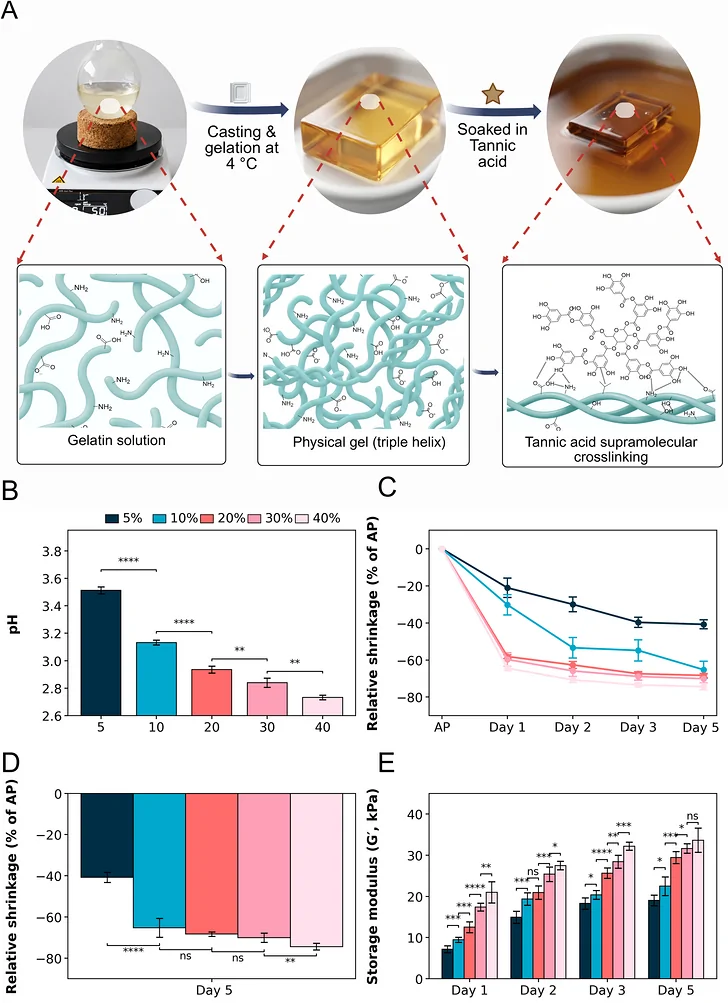
Tannic acid treatment induces rapid volume contraction and progressive mechanical reinforcement of gelatin hydrogels. (A) Photographic and schematic overview of physical hydrogel formation and tannic acid treatment. The upper row shows photographs of the actual gelatin solution, physically gelled sample, and tannic‐acid‐treated hydrogel, while the lower row illustrates the corresponding network organization. Cooling of the gelatin solution produces a physical network through triple‐helix formation and chain entanglement. Subsequent immersion in tannic acid induces supramolecular association, network compaction, and macroscopic contraction. (B) Measured pH of aqueous tannic acid solutions containing 5–40 wt.% tannic acid. (C) Relative volumetric shrinkage during tannic acid treatment. (D) Endpoint volumetric shrinkage after 5 days. (E) Storage modulus (G′) as a function of treatment time, showing concentration‐dependent stiffening. Data are presented as mean ± standard deviation (SD).. Statistical significance was determined by two‐way analysis of variance (ANOVA) followed by Tukey’s honestly significant difference (HSD) post hoc test (ns, not significant; ^*^ *p* < 0.05, ^**^ *p* < 0.01, ^***^ *p* < 0.001, ^****^ *p* < 0.0001.

The structural evolution proceeds in two distinct stages. Within the first 24 h, the hydrogel underwent rapid volume contraction to approximately 22% of its initial volume, indicating substantial water loss (Figure 1C and Figure S1A). This was followed by a slower evolution of the viscoelastic properties during continued incubation in the absence of any additional covalent crosslinking. Although the macroscopic volume remained stable after the first day, the internal network continued to reorganize. Consistent with this continued network reorganization, the final viscoelastic state in Figure 1E depended strongly on tannic acid concentration. After 5 days, G′ increased from 18.9 kPa at 5 wt.% tannic acid to 23.0, 29.3, 31.6, and 32.9 kPa at 10, 20, 30, and 40 wt.%, respectively. In parallel, tan δ increased from 0.274 at 5 wt.% to 0.321, 0.326, 0.424, and 0.438 at 10, 20, 30, and 40 wt.%, respectively. Thus, increasing the tannic acid concentration not only stiffened the compacted network but also increased its dissipative character, consistent with the formation of a progressively more constrained yet dynamically rearrangeable supramolecular phase. The loss modulus (G″) increased from approximately 6 kPa on Day 1 to approximately 14 kPa by Day 5 (Figure S1B), indicating enhanced viscous energy dissipation. The progressive increases in G″ and tan δ (approximately 0.30–0.45) indicate the development of a more dissipative viscoelastic network dominated by non‐covalent interactions [21, 22]. This behavior is consistent with ongoing rearrangement of supramolecular interactions, potentially involving additional tannic‐acid–gelatin associations, chain reorganization, or partial restructuring of helix‐derived junctions, although the precise molecular origin cannot be resolved from rheology alone.

Despite this pronounced compaction and increased dissipation, the purely physical Tan‐GelPHy network remains intrinsically metastable in aqueous environments [25, 30]. Upon transfer to deionized water, Tan‐GelPHy progressively re‐swelled and exceeded its initial volume by approximately 50% at Day 5. The initially compact brown hydrogel also became markedly paler and opaque, as shown in Figure S1C. This visual change is consistent with relaxation of the purely physical network and possible loss or redistribution of physically associated tannic acid, but is not interpreted as a quantitative measure of tannic acid release. The structural relaxation was accompanied by a marked reduction in viscoelasticity, with G′ decreasing to 5.23 kPa, as shown in Figure S1D. These observations highlight the metastability of purely supramolecular tannic‐acid–gelatin networks and motivate the introduction of covalent crosslinking to stabilize the compact architecture.

### 2.2 Acid‐Triggered Bis‐Oxazoline Chemistry Enables In Situ Crosslinking of Compacted Gelatin

The second key challenge was stabilizing this system by covalent crosslinking [11, 12, 13]. Traditional crosslinking strategies for proteins often fail at acidic pH because protonation of nucleophilic amino groups reduces their reactivity [13, 15]. Therefore, most strategies are designed to promote crosslinking at neutral or basic pH. Conventional carbodiimide‐based coupling reagents such as 1‐ethyl‐3‐(3‐dimethylaminopropyl) carbodiimide require slightly acidic pH (≈4.5); however, this limits the reactivity of amino residues [12, 15]. Thus, active ester strategies are often incorporated by adding additives such as N‐hydroxysuccinimide or 1‐hydroxybenzotriazole to overcome this limitation. Other reagents, such as genipin, also typically require neutral to alkaline conditions (pH 7–9) to function effectively [13, 15]. However, increasing the pH of the tannic acid‐compacted gels induces immediate decompaction and swelling, thereby disrupting the compact architecture that we aim to preserve.

To address this constraint, we identified 2,2′‐bis(2‐oxazoline) (herein termed “bis‐oxazoline”) as a suitable candidate [33, 34]. Unlike amine‐targeting chemistries, bis‐oxazoline reacts with carboxylic acid groups through an acid‐promoted ring‐opening mechanism [33, 34]. The applicability of oxazoline–carboxylic acid chemistry to gelatin‐based networks has previously been demonstrated using poly(2‐isopropenyl‐2‐oxazoline) [35]. The proposed reaction pathway is shown in Scheme 1. In our system, bis‐oxazoline was pre‐embedded in the gelatin matrix prior to tannic acid treatment. Upon acidification of the scaffold during tannic acid exposure, the oxazoline groups become activated and react with carboxylic acid residues on the gelatin chains through the proposed ring‐opening pathway, resulting in bis‐oxazoline‐mediated covalent linkages [33, 34].

**SCHEME 1:**
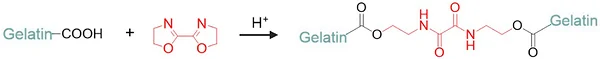
Proposed acid‐promoted ring‐opening reaction between free gelatin carboxylic acid residues and 2,2′‐bis(2‐oxazoline) crosslinker to obtain diester crosslinked gelatin hydrogels.

The intrinsic pH of the tannic‐acid‐treated hydrogels (≈2.7) is sufficiently acidic to support this reaction pathway. In addition, pronounced tannic‐acid‐mediated compaction, corresponding to an approximately 80% volume loss, increases the effective local concentration of reactive groups and is therefore expected to favor intermolecular crosslinking [21, 22]. To distinguish acidification from tannic‐acid‐induced physical compaction, the bis‐oxazoline‐crosslinked physical gelatin hydrogel (Oxo‐GelPHy) was also immersed in an HCl solution adjusted to pH 2.7 without tannic acid. The pH‐matched HCl treatment did not reproduce the pronounced dimensional contraction observed during tannic acid treatment. Instead, the samples showed substantial swelling and reduced dimensional stability. These findings show that acidification alone is insufficient to generate the compact architecture and support distinct roles for acid activation and tannic‐acid‐induced physical compaction (Figure S4). As illustrated in Figure 2, tannic acid also provides supramolecular interactions that help maintain structural integrity under acidic conditions, thereby allowing covalent crosslinking to proceed without dissolution of the hydrogel network [21, 22].

**FIGURE 2:**
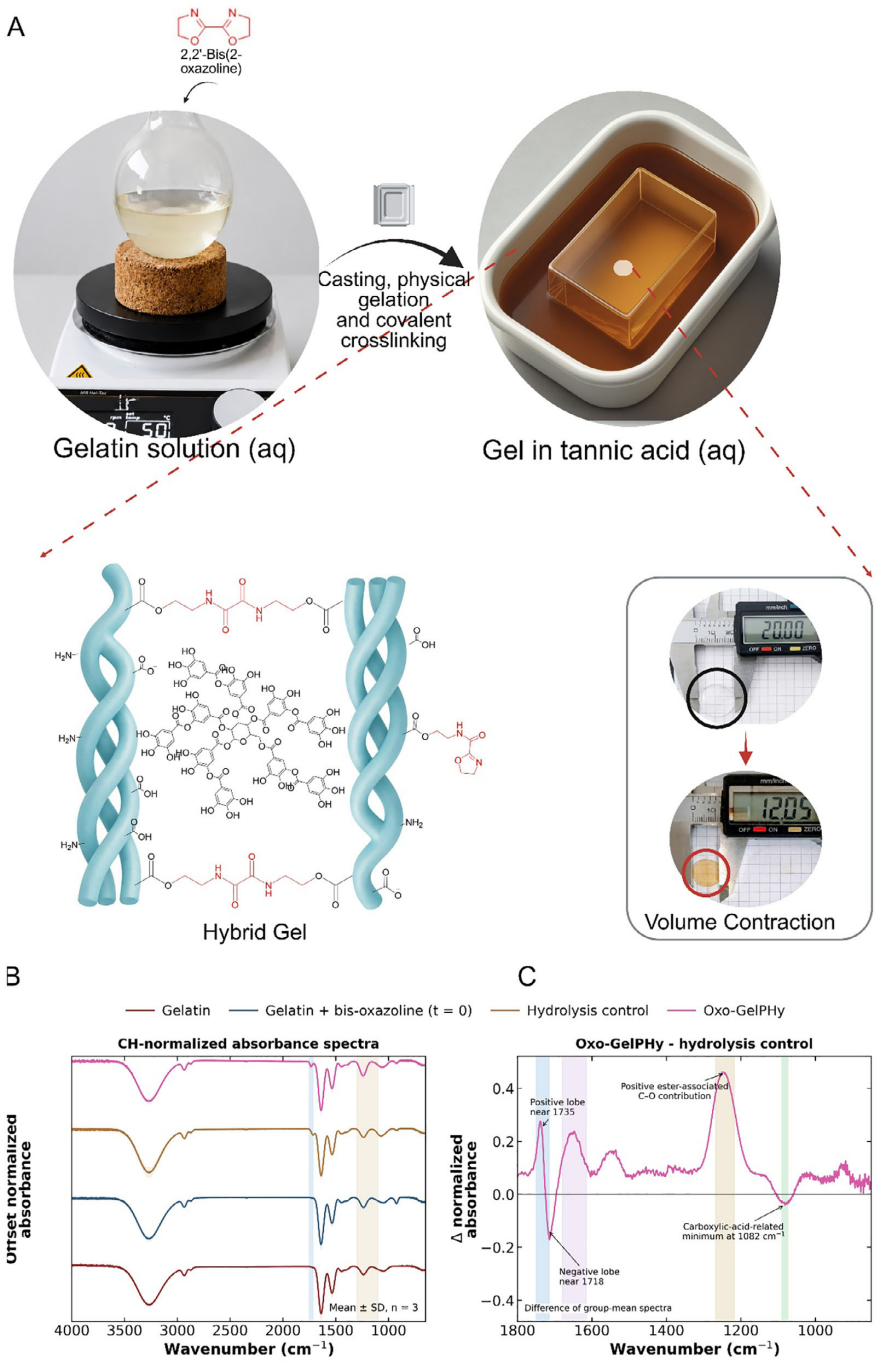
Photographic, schematic, and spectroscopic overview of TanOxo‐Gel formation. (A) Bis‐oxazoline‐containing gelatin is cast, physically gelled, and immersed in tannic acid, inducing supramolecular compaction and bis‐oxazoline‐mediated covalent stabilization. (B) C‐H‐normalized attenuated total reflection Fourier‐transform infrared spectroscopy (ATR‐FTIR) absorbance spectra of unmodified gelatin, gelatin with bis‐oxazoline immediately after mixing (*t* = 0), the acid‐mediated hydrolysis control, and Oxo‐GelPHy. (C) Difference spectrum calculated as Oxo‐GelPHy minus the hydrolysis control, showing positive ester‐associated contributions near 1735 and 1242 cm^−1^ and negative carboxylic‐acid‐associated contributions near 1718 and 1082 cm^−1^.

To provide spectroscopic support for bis‐oxazoline‐mediated chemical network formation, attenuated total reflection Fourier‐transform infrared spectroscopy (ATR‐FTIR) analysis was performed using unmodified gelatin, gelatin with 2,2′‐bis(2‐oxazoline) immediately after mixing (*t* = 0), an acid‐mediated bis‐oxazoline hydrolysis control, and the washed Oxo‐GelPHy network (Figure 2B,C). Detailed comparisons of the carbonyl and ester‐associated C─O regions, targeted carbonyl‐band fitting, and the descriptive C─O/C─H fitted‐area analysis are presented in Figure S5A–D. The C─H‐normalized spectra retained the characteristic gelatin features while revealing distinct changes in the carbonyl and ester‐associated C─O regions of Oxo‐GelPHy. Targeted local fitting identified an ester‐associated carbonyl‐band maximum at 1735 cm^−1^ for Oxo‐GelPHy, compared with a carbonyl‐band maximum at 1718 cm^−1^ for the acid‐mediated hydrolysis control (Figure S5A,C). The lower‐wavenumber carbonyl band in the hydrolysis control is consistent with carboxylic‐acid‐containing hydrolysis products, whereas the higher‐wavenumber carbonyl band in Oxo‐GelPHy is consistent with ester formation. Consistently, the difference spectrum, calculated as the mean C─H‐normalized Oxo‐GelPHy spectrum minus the mean C─H‐normalized hydrolysis‐control spectrum, showed positive difference features near 1735 and 1242 cm^−1^ and negative difference features near 1718 and 1082 cm^−1^. The positive features reflect ester‐associated carbonyl and C─O contributions in Oxo‐GelPHy. The negative feature near 1718 cm^−1^ reflects the stronger carboxylic‐acid‐associated carbonyl contribution in the hydrolysis control, whereas the minimum near 1082 cm^−1^ is consistent with a carboxylic‐acid‐related C─O contribution. These negative features represent relative spectral differences and do not correspond to negative absorbance. The ester‐associated C─O contribution near 1242 cm^−1^ was further supported by the regional comparison in Figure S5B. Consistently, the descriptive C─O/C─H fitted‐area ratio increased to 0.395 for Oxo‐GelPHy, compared with 0.213–0.251 for the control conditions (Figure S5D). Together, these control‐resolved spectral changes distinguish the washed Oxo‐GelPHy network from the initially mixed and hydrolyzed bis‐oxazoline‐containing controls and provide spectroscopic evidence for bis‐oxazoline‐mediated covalent network formation.

As complementary macroscopic evidence, Gel‐PHy, Oxo‐GelPHy, Tan‐GelPHy, and TanOxo‐Gel were compared before and after incubation at 60°C for 48 h (Figure S6). Gel‐PHy and Tan‐GelPHy showed pronounced loss of macroscopic integrity, whereas Oxo‐GelPHy and TanOxo‐Gel retained visually identifiable gel material. This observation provides complementary evidence of thermally persistent network stabilization and is not interpreted as independent structural proof of a specific covalent linkage.

### 2.3 Optimization of Covalent Crosslinking Conditions to Obtain Structurally Stable Materials

We next sought to identify the optimal bis‐oxazoline concentration that most effectively stabilizes the compact architecture under these acidic conditions. Since bis‐oxazoline is a bifunctional crosslinker, the oxazoline‐to‐carboxylic‐acid molar ratio is expected to critically influence productive network formation [33]. In principle, full bifunctional bridging would correspond to one bis‐oxazoline molecule reacting with two carboxylic acid groups. However, oxazoline groups are known to be susceptible to acid‐mediated hydrolysis, and competing non‐bridging reactions can further reduce the efficiency of productive intermolecular coupling [33, 36]. Because swelling in crosslinked polymer networks is strongly coupled to network density, the volumetric response was used here as a key readout of effective crosslink formation [37]. We therefore prepared gelatin hydrogels preloaded with 0.5–2.0 equivalents of bis‐oxazoline relative to the available gelatin carboxylic acid groups and systematically evaluated their volumetric response, swelling behavior, and rheological properties as a function of crosslinker loading in order to identify the most effective formulation within the screened range. These experiments were carried out at 4°C for 48 h, as such low temperatures are expected to promote maximal renaturation of gelatin chains to obtain robust chemically crosslinked gels [11, 12, 13, 14, 15]. To allow complete crosslinking, the samples were further incubated at room temperature for an additional 5 days to obtain chemically stabilized gels, hereafter termed Oxo‐GelPHy, corresponding to bis‐oxazoline‐crosslinked physical gelatin hydrogels prepared in the absence of tannic acid.

As shown in Figure 3A (left panel), Oxo‐GelPHy exhibited progressive and strongly formulation‐dependent swelling during the 21‐day aqueous stability study. The lowest‐loading formulations, containing 0.50 and 0.75 eq bis‐oxazoline, lost macroscopic integrity by Day 21 and are therefore indicated by circled X symbols rather than quantitative Day 21 swelling values (Figure 3A). At Day 21, the remaining formulations exhibited relative swelling values of 225.0% at 1.0 eq, 185.0% at 1.25 eq, 78.0% at 1.5 eq, 155.0% at 1.75 eq, and 234.6% at 2.0 eq. Among the screened formulations, 1.50 eq bis‐oxazoline provided the lowest Day 21 swelling and the greatest overall dimensional stability within the Oxo‐GelPHy series. These findings identify an intermediate bis‐oxazoline loading as the most effective condition within the investigated range for stabilizing the chemically crosslinked gelatin network.

**FIGURE 3:**
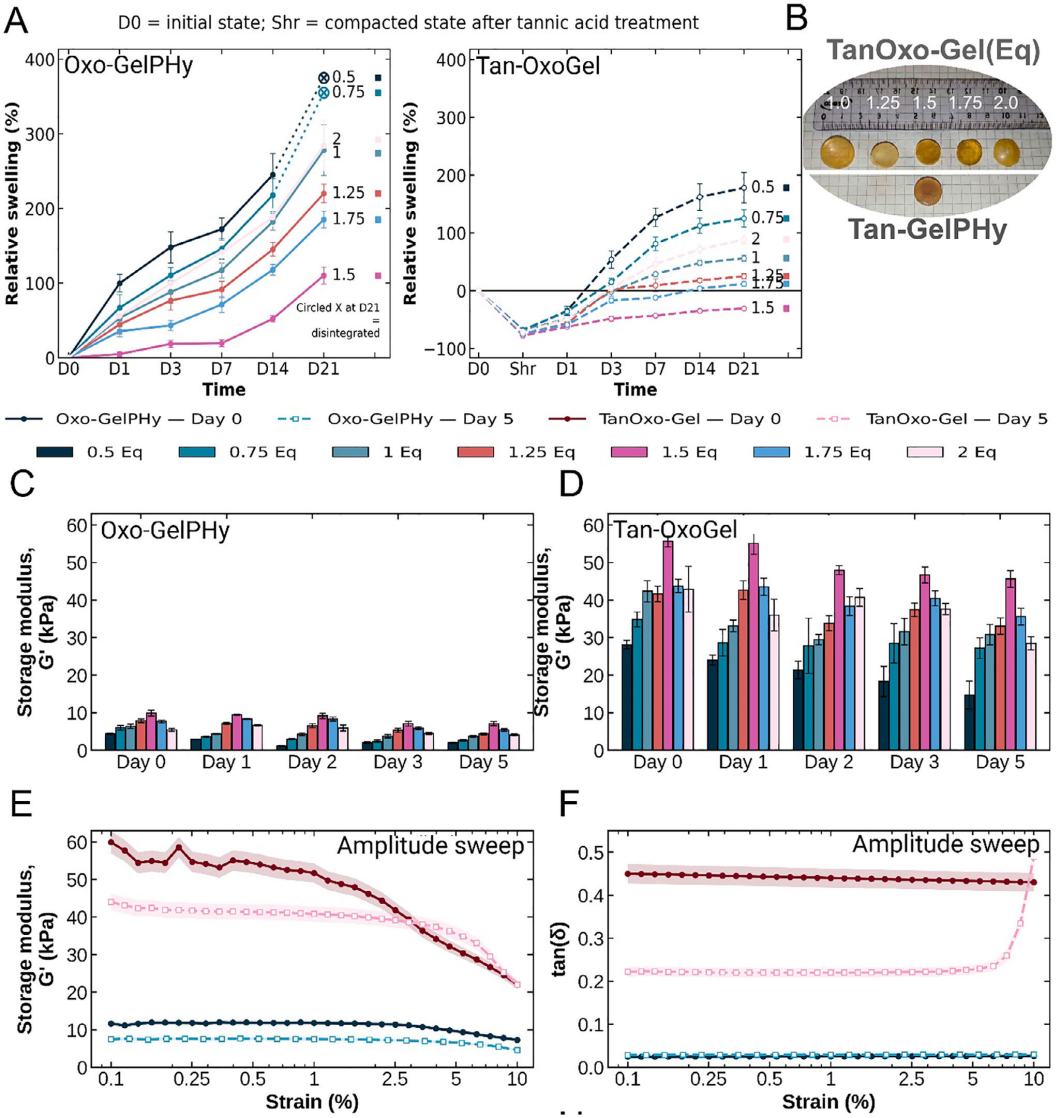
Bis‐oxazoline‐mediated chemical network formation stabilizes tannic‐acid‐compacted gelatin hydrogels and enables tunable mechanical properties. (A) Time‐dependent relative volumetric change during immersion in water. Left: Oxo‐GelPHy relative to the initial state (D0). The 0.50 and 0.75 eq formulations lost macroscopic integrity by Day 21 and are indicated by circled X symbols; consequently, no quantitative Day 21 swelling values are reported for these formulations. Right: relative volumetric change of TanOxo‐Gel referenced to the compacted state after tannic acid treatment (Shr) relative to D0 and subsequent aqueous incubation through Day 21. (B) Representative photographs of equilibrated TanOxo‐Gel samples prepared with 1.0–2.0 eq bis‐oxazoline, together with the Tan‐GelPHy control. (C) Storage modulus (G′) of Oxo‐GelPHy and (D) G′ of TanOxo‐Gel as a function of bis‐oxazoline equivalent and immersion time. Data are presented as mean ± standard deviation. Panels E and F show the rheological characterization of the optimized 1.50 eq formulation at 25°C: (E) amplitude sweeps of G′ as a function of strain for Oxo‐GelPHy and TanOxo‐Gel at Day 0 and Day 5, and (F) the corresponding loss tangent (tan δ) values for the same formulations and immersion times.

To evaluate the effect of tannic acid treatment, the same formulations were subsequently immersed in a 30 wt.% tannic acid bath at pH 2.7 and 4°C for an additional 48 h. This cold‐diffusion phase allowed tannic acid to penetrate the matrix and initiate compaction while maintaining the stability of the triple‐helix junctions and limiting the covalent reaction rate [11, 12, 13, 14, 15, 21, 22]. The samples were then transferred to room temperature for an additional 72 h to complete bis‐oxazoline‐mediated chemical network formation and obtain TanOxo‐Gel, corresponding to tannic‐acid‐compacted, bis‐oxazoline‐crosslinked physical gelatin hydrogels.

As shown in Figure 3A (right panel), all formulations contracted after tannic acid treatment. The relative volumetric change at the compacted state (Shr) ranged from −69.0% at 0.50 eq and −67.8% at 0.75 eq to −74.8% at 1.25 eq, reaching a maximum compaction of −77.8% at 1.50 eq. Following tannic acid treatment, the aqueous stability of all TanOxo‐Gel formulations was evaluated in water for 21 days. All TanOxo‐Gel formulations retained macroscopic integrity throughout the study, in contrast to the disintegration observed for the lowest‐loading Oxo‐GelPHy controls. At Day 21, the 0.50 eq formulation showed a relative volumetric change of 178.0%. Increasing the bis‐oxazoline loading to 0.75 and 1.00 eq reduced the expansion to 125.0% and 56.0%, respectively. The 1.25 eq formulation showed a relative volumetric change of 24.9%, whereas the 1.50 eq formulation most effectively retained the compacted state, with a relative volumetric change of −31.0%. At 1.75 and 2.00 eq, the corresponding values were 11.7% and 88.3%, respectively. The 1.50 eq formulation showed only a limited additional change between Day 14 and Day 21, from −35.0% to −31.0%, indicating that it had approached a stable dimensional plateau. Visual inspection of the immersion media revealed a clear formulation‐dependent trend in the retention of tannic‐acid‐derived species. Visible brown coloration was observed in the medium surrounding the Tan‐GelPHy control and the low‐bis‐oxazoline TanOxo‐Gel formulations at 0.50 and 0.75 eq. The coloration progressively diminished with increasing bis‐oxazoline loading and was not visually detectable for the optimized 1.50 eq formulation. This trend is consistent with increasingly effective retention of tannic‐acid‐derived species within the stabilized gelatin network, potentially through oxidation‐associated polymerization and/or covalent interactions [22, 28]. Although this qualitative observation does not constitute a quantitative cumulative tannic‐acid‐leaching assay, the absence of visible coloration for the 1.50 eq formulation supports that tannic‐acid‐derived species were largely retained during aqueous immersion. Figure 3B provides a visual comparison of TanOxo‐Gel formulations prepared with 1.00–2.00 eq bis‐oxazoline. The larger dimensions and more opaque, whitish appearance of the more swollen gels are consistent with greater aqueous expansion and reduced structural control. Taken together with the rheological results presented below, these observations indicate that 1.50 eq provides the most effective crosslinking condition within the screened range for stabilizing the compact architecture, whereas higher loadings resulted in reduced crosslinking efficiency, potentially because competing oxazoline hydrolysis and monosubstitution increased the proportion of non‐bridging reactions [33, 36].

### 2.4 Rheology Identifies an Optimal Intermediate Bis‐Oxazoline Loading

As shown in Figure 3D, the storage modulus (G′) depended strongly on crosslinker loading. Across the full 0.5‐2.0 eq screening range, the retained modulus after 5 days increased from 14.7 kPa at 0.5 eq to 27.2 kPa at 0.75 eq, 30.8 kPa at 1.0 eq, and 33.1 kPa at 1.25 eq, before reaching a maximum of 45.6 kPa at 1.5 eq and then decreasing again to 35.6 kPa at 1.75 eq and 28.5 kPa at 2.0 eq. Thus, the lower‐loading formulations remained insufficiently stabilized, whereas higher loading reduced network efficiency. Within this screening series, 1.5 eq gave the highest initial and retained modulus, reaching 55.7 kPa initially and remaining at 45.6 kPa after immersion. By contrast, further increasing the loading reduced stiffness, consistent with non‐bridging reactions and network defects rather than more effective interchain coupling [33, 34, 36]. This storage modulus range places the material within a mechanically relevant regime for hydrogel‐based biomaterials, for which mechanical properties are an important scaffold design parameter [38].

The importance of combining tannic acid‐mediated compaction with covalent crosslinking is evident from the control systems. Pristine 10 wt.% gelatin hydrogels exhibit a storage modulus of 3–5 kPa, consistent with reported values for uncrosslinked physical gelatin gels [11, 12, 13, 14, 15]. Under acidic conditions (pH 2), the physical gelatin network softens to approximately 0.9 kPa, consistent with acid‐induced weakening of the non‐covalent network [32]. Even in the chemically crosslinked control without tannic acid (Oxo‐GelPHy), the same non‐monotonic dependence on bis‐oxazoline loading was observed, but at substantially lower absolute moduli. After 5 days of immersion, the 1.25, 1.5, and 1.75 eq Oxo‐GelPHy formulations retained G′ values of 4.4, 7.1, and 5.4 kPa, respectively, indicating that the crosslinking chemistry is operative even in the absence of tannic acid‐mediated compaction, but that the resulting network remains weak and relaxes progressively in water. In contrast, the corresponding tannic acid‐compacted hydrogels retained markedly higher moduli of 33.1, 45.6, and 35 kPa at 1.25, 1.5, and 1.75 eq, respectively. This trend is also consistent with previous reports showing that incorporation of tannic acid can enhance crosslinking efficiency and network stabilization in gelatin‐based hydrogels [39].

To further characterize the viscoelastic response of the optimized 1.5 eq formulation, amplitude sweeps were performed for TanOxo‐Gel and the corresponding Oxo‐GelPHy control at Day 0 and after 5 days of immersion (Figure 3E,F). TanOxo‐Gel exhibited substantially higher storage modulus values than Oxo‐GelPHy throughout the investigated strain range (Figure 3E). Within the low‐strain region, TanOxo‐Gel showed a G′ of approximately 60 kPa at Day 0 and 42 kPa at Day 5, whereas the corresponding Oxo‐GelPHy values were approximately 11 and 7 kPa, respectively. For both hydrogel systems, 0.5% strain remained within the linear viscoelastic region and was therefore selected for the subsequent frequency sweeps presented in Figure S3C.

The corresponding loss‐modulus data further demonstrated the pronounced difference in dissipative response between the two networks (Figure S3C). During the amplitude sweeps, TanOxo‐Gel exhibited G″ values of approximately 24–27 kPa within the low‐strain region at Day 0, decreasing to approximately 8–9 kPa after 5 days of immersion. In contrast, the corresponding G″ values of Oxo‐GelPHy remained below approximately 0.5 kPa. The frequency sweeps performed at 0.5% strain showed the same distinction: TanOxo‐Gel maintained G″ values of approximately 23–24 kPa at Day 0 and 7–8 kPa at Day 5 across the investigated frequency range, whereas Oxo‐GelPHy exhibited substantially lower, sub‐kPa G″ values. These results demonstrate that the tannic‐acid‐compacted network retained a markedly greater viscous and energy‐dissipative contribution both before and after aqueous equilibration.

Importantly, covalent reinforcement did not eliminate viscoelasticity. As shown in Figure 3F, the loss factor (tan δ) of the optimized 1.5 eq TanOxo‐Gel formulation decreased from approximately 0.45 in the as‐prepared condition to approximately 0.22 after 5 days of immersion, whereas Oxo‐GelPHy remained predominantly elastic, with tan δ values of approximately 0.02–0.03. This difference indicates that the tannic acid‐containing network retained a substantially larger dissipative contribution after equilibration [21, 26]. Together, the G″ and tan δ results demonstrate that the optimized TanOxo‐Gel network retained a pronounced dissipative viscoelastic component after immersion. Taken together, the swelling and storage‐modulus screening data identify 1.5 eq as the rheological optimum. At this composition, the network most effectively suppresses re‐swelling, exhibits the highest and best‐retained G′ after immersion, and retains a measurable dissipative viscoelastic component.

### 2.5 Dual Supramolecular‐Covalent Networks Deliver Exceptional Tensile Strength, Extensibility, and Toughness

Based on the combined swelling and rheological analyses in Section 2.3, the 1.5 eq formulation was identified as the optimized TanOxo‐Gel composition. To determine how deviations from this rheological optimum effect large‐strain mechanical performance, the neighboring 1.25 and 1.75 eq formulations were included for comparison, as shown in Figure 4A–C. As shown in Figure 4B, TanOxo‐Gel (1.5 eq) exhibited a characteristic J‐shaped stress‐strain curve, with an initially compliant low‐strain regime followed by pronounced strain hardening at higher strain. In the low‐strain regime, the response likely reflects both elastic deformation of the network and viscoelastic dissipation associated with progressive dissociation and rearrangement of reversible tannic acid‐gelatin interactions within the supramolecular phase [21, 22, 25, 26]. These interactions may include hydrogen bonding between phenolic hydroxyl groups of tannic acid and the gelatin backbone, hydrophobic contacts between aromatic rings and nonpolar amino acid residues, and π‐π interactions between tannic acid galloyl groups [21, 22, 25, 26]. Their progressive disruption enables chain sliding and energy dissipation, whereas the remaining network elasticity maintains mechanical continuity. At higher strain, load transfer increasingly shifts to aligned gelatin chains and the bis‐oxazoline‐mediated covalent network, giving rise to the observed strain hardening. The tensile mechanical response of TanOxo‐Gel therefore reflects synergistic contributions from the supramolecular and covalent networks.

**FIGURE 4:**
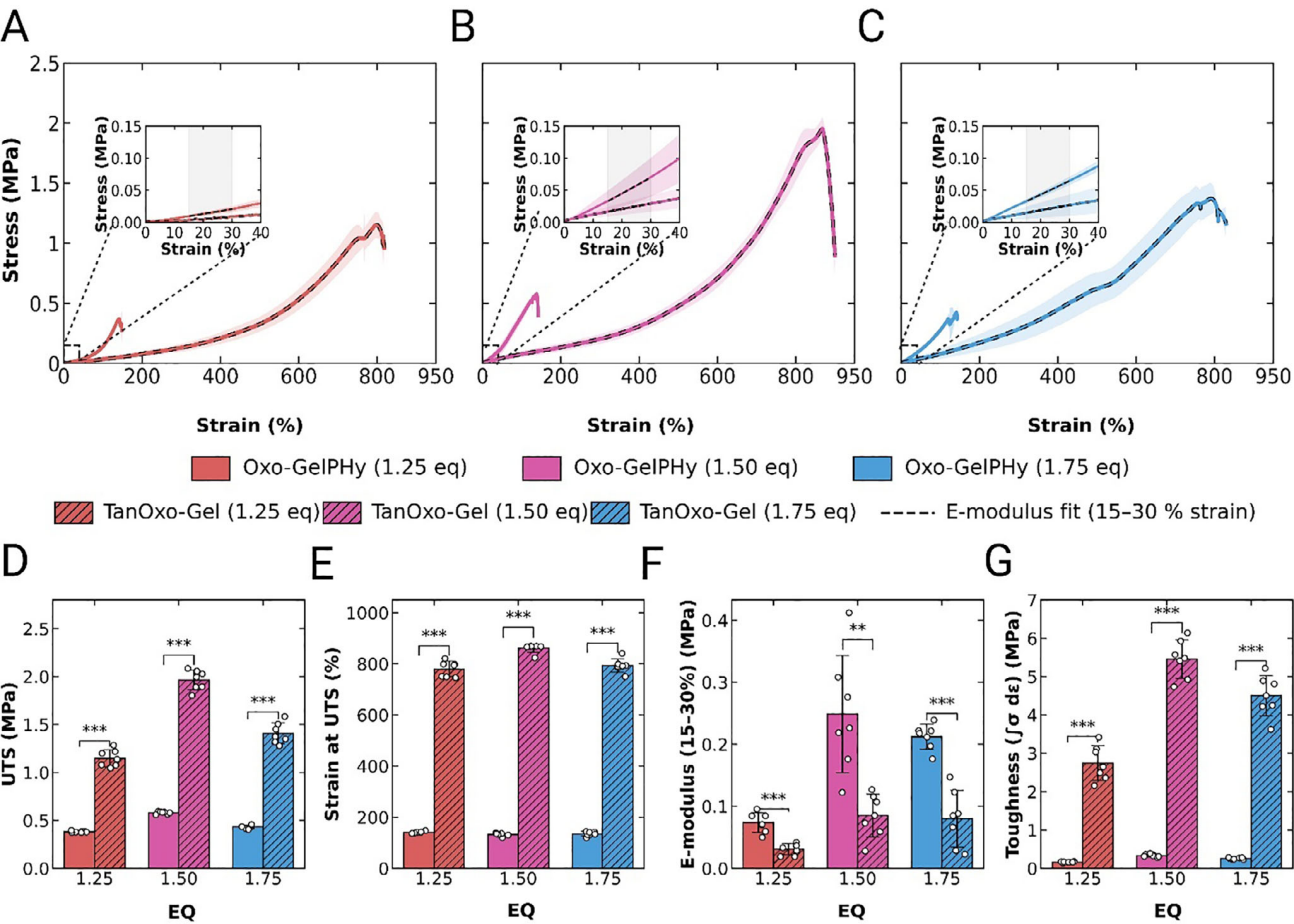
Large‐strain tensile mechanics of TanOxo‐Gel compared with Oxo‐GelPHy. (A–C) Representative engineering stress‐strain curves of Oxo‐GelPHy and TanOxo‐Gel at bis‐oxazoline loadings of 1.25 eq (A), 1.5 eq (B), and 1.75 eq (C). Insets show the low‐strain region used to determine the elastic modulus from linear fits over 15%–30% strain. (D) Ultimate tensile strength (UTS). (E) Strain at UTS. (F) Elastic modulus determined from the 15%–30% strain region. (G) Toughness calculated as the integrated area under the stress‐strain curve. Data are presented as mean ± standard deviation. Statistical significance was determined by two‐way analysis of variance (ANOVA) with Tukey’s honestly significant difference (HSD) post hoc test (ns, not significant; ^*^ *p* < 0.05, ^**^ *p* < 0.01, ^***^ *p* < 0.001, ^****^ *p* < 0.0001).

TanOxo‐Gel (1.5 eq) exhibited an ultimate tensile strength of 1.97 MPa, a strain at maximum stress of 896%, and a toughness of 5.46 MJ m^−3^ (Figure 4D,E,G). Together, these data show that the material combines MPa‐level strength, high extensibility, and substantial energy absorption. By contrast, the corresponding Oxo‐GelPHy control remained markedly weaker and less extensible, failing below 150% strain, with an ultimate tensile strength below 0.5 MPa and a toughness below 0.3 MJ m^−3^ (Figure 4D,E,G). The apparent modulus determined from the 15%–30% strain region was lower for TanOxo‐Gel than for Oxo‐GelPHy (Figure 4F), indicating that tannic acid‐mediated compaction does not increase the low‐strain stiffness of the network, but instead promotes a tougher and more extensible large‐strain response.

Mechanical performance also depended strongly on crosslinker loading. The 1.5 eq formulation exhibited the highest tensile strength and toughness, while 1.25 and 1.75 eq showed lower values (Figure 4D,G). A similar trend was observed for strain at maximum stress, with 1.5 eq showing the greatest extensibility among the screened formulations (Figure 4E). By contrast, the low‐strain modulus followed a different pattern, with Oxo‐GelPHy exhibiting higher values in the 15%–30% strain regime (Figure 4F). Taken together, these results show that decoupling supramolecular compaction from covalent crosslinking enables formation of a hybrid hydrogel that combines a robust load‐bearing framework with high large‐strain deformability. This design is consistent with established tough‐hydrogel strategies based on the integration of elastic and dissipative network components [16, 17, 18, 19, 20, 40, 41], while uniquely leveraging coacervation‐driven compaction to enhance network efficiency [21, 22, 25, 26, 42]. The favorable balance of strength, extensibility, and toughness observed for TanOxo‐Gel therefore motivated further evaluation of cyclic hysteresis and energy dissipation.

In situ optical imaging during deformation revealed localized whitening in the gauge region immediately prior to failure (Videos S1–S3), a phenomenon commonly associated with deformation‐induced structural heterogeneity and energy dissipation [18]. This feature was absent in Oxo‐GelPHy under identical testing conditions (Videos S4–S6), indicating a distinct deformation and failure process.

### 2.6 Reversible Tannic Acid Gelatin Interactions Impart High Hysteresis and Sustained Cyclic Dissipation

Based on the combined swelling, rheological, and tensile analyses in Sections 2.3 and 2.4, the 1.5 eq formulation was selected as the optimized composition. Unless otherwise stated, TanOxo‐Gel hereafter refers to this optimized formulation. Oxo‐GelPHy and TanOxo‐Gel showed markedly different cyclic mechanical responses, revealing the contribution of the tannic acid‐rich supramolecular phase to energy dissipation. At the target strain corresponding to 75% of the formulation‐specific ultimate elongation at break, TanOxo‐Gel reached a peak stress of 1.64 MPa (*n* = 3), whereas Oxo‐GelPHy reached only 0.394 MPa (*n* = 3), as shown in Figure 5C,F. This marked difference highlights the contribution of tannic acid‐mediated compaction to the load‐bearing capacity of the network. While the high peak stress reflects the mechanically robust, covalently stabilized structure of TanOxo‐Gel, the pronounced hysteresis observed during cyclic loading is consistent with reversible supramolecular interactions within the tannic acid‐rich phase that contribute to energy dissipation [43].

**FIGURE 5:**
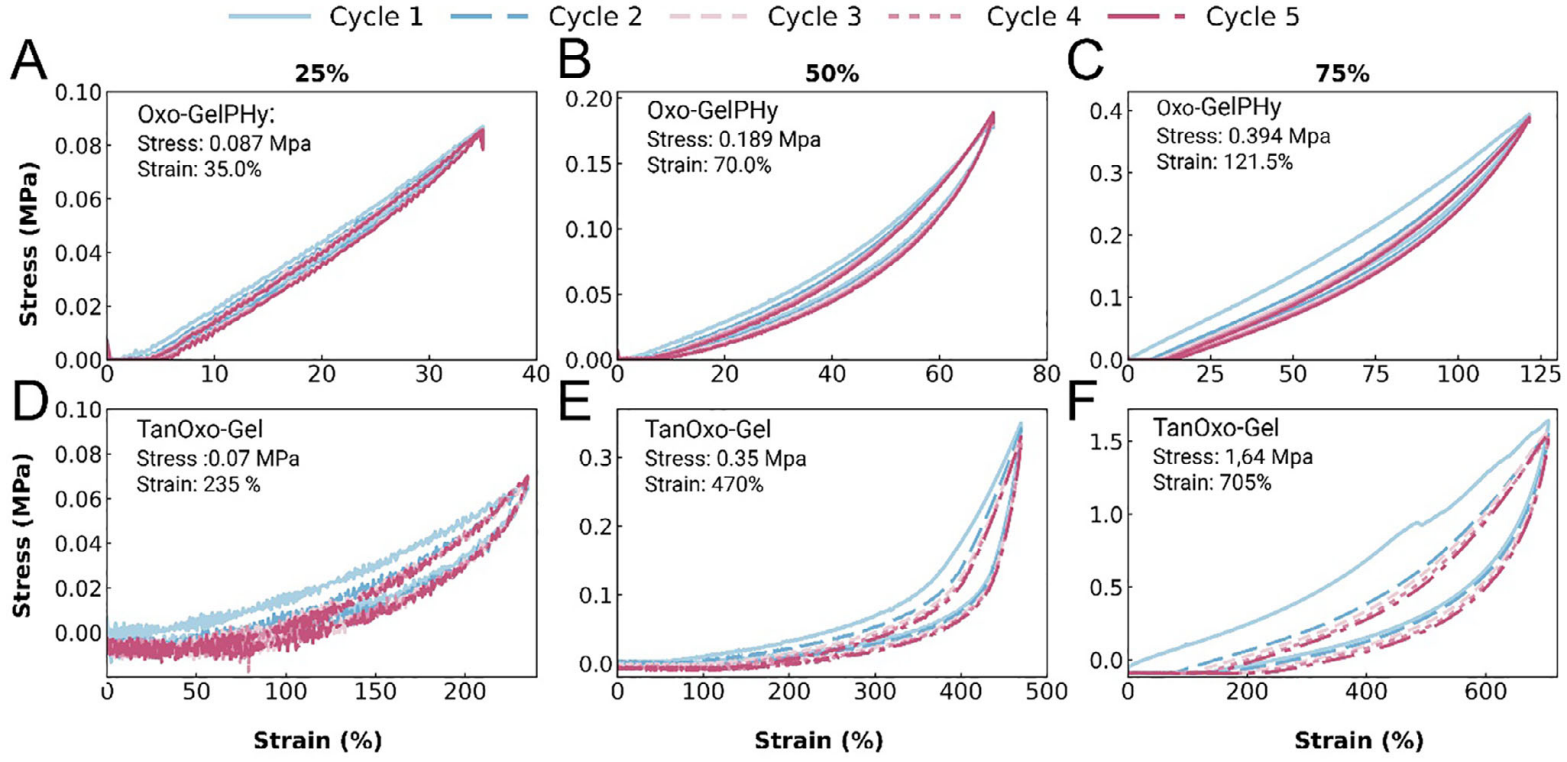
Cyclic tensile response of TanOxo‐Gel and Oxo‐GelPHy. (A–C) Representative cyclic engineering stress–strain curves of Oxo‐GelPHy obtained at peak strains corresponding to 25%, 50%, and 75% of the formulation‐specific ultimate elongation at break. (D–F) Representative cyclic engineering stress–strain curves of TanOxo‐Gel obtained at peak strains corresponding to 25%, 50%, and 75% of the formulation‐specific ultimate elongation at break. Separate specimens were used for each target strain. Because the formulations exhibited different ultimate elongations at break, the corresponding absolute peak strains differed between the hydrogel systems. Annotated values indicate the peak stress and absolute strain reached during each cycle. The corresponding dissipated‐energy analysis is presented in Figure S8C.

The contribution of the tannic acid‐rich supramolecular phase was further assessed by comparison with Oxo‐GelPHy across the full cyclic loading series (Figure 5A–F). In contrast to TanOxo‐Gel (Figure 5D–F), Oxo‐GelPHy (Figure 5A–C) displayed a more nearly linear elastic response with negligible hysteresis, indicating markedly lower energy dissipation. In the absence of the tannic acid‐rich supramolecular network, the material exhibited predominantly elastic deformation and failed at substantially lower strain.

Following the large first‐cycle dissipation, TanOxo‐Gel exhibited pronounced Mullins‐like softening, defined here as a lower stress response and reduced hysteresis in subsequent loading cycles relative to the first cycle (Figure 5D–F) [44]. From Cycles 2–5, the stress–strain response approached a more repeatable path with substantially reduced hysteresis. This behavior suggests that part of the initial energy dissipation is associated with network rearrangement during the first loading cycle, while the covalent network preserves overall connectivity during repeated deformation. Importantly, the hysteretic response was not assessed only qualitatively from the cyclic stress–strain loops. Instead, the dissipated energy density, U_diss_ was quantified by numerical integration of the area enclosed by each loading–unloading loop. The results are reported for Cycle 1, the mean of Cycles 2–5, Cycle 5, and cumulatively over the complete five‐cycle loading protocol in Figure S8C. For the optimized TanOxo‐Gel formulation tested at 75% of its formulation‐specific ultimate elongation at break, the first‐cycle dissipated energy density reached 4.21 MJ m^−3^, compared with 0.219 MJ m^−3^ for Oxo‐GelPHy. The mean dissipation over Cycles 2–5 remained substantial at 1.18 MJ m^−3^ for TanOxo‐Gel, compared with 0.043 MJ m^−3^ for Oxo‐GelPHy, while the corresponding Cycle 5 values were 1.47 and 0.149 MJ m^−3^, respectively. Over the complete five‐cycle protocol, the cumulative dissipated energy density reached 10.11 MJ m^−3^ for TanOxo‐Gel, compared with 0.436 MJ m^−3^ for Oxo‐GelPHy. The corresponding cyclic stress–strain curves for the different formulations and target strain levels are shown in Figure S8A,B, and the corresponding cyclic deformation videos are provided as Videos S7–S9.

These cyclic responses are consistent with the interplay between reversible supramolecular tannic acid–gelatin interactions and the permanent covalent network [43]_._ During the first loading cycle, disruption and rearrangement of reversible non‐covalent interactions contribute substantially to the large hysteresis, whereas the covalent network maintains structural continuity. After this initial rearrangement, the stress–strain response becomes more reproducible, with reduced hysteresis in subsequent cycles. Taken together, these features are consistent with multiscale energy dissipation in native collagenous tissues [6].

### 2.7 The Compact Covalently Stabilized Matrix Preserves Cytocompatibility

Although the mechanical performance of TanOxo‐Gel (1.5 eq) was promising, the relatively high tannic acid content of the material could raise concerns regarding cytocompatibility [45, 46]. To assess the effect of tannic acid‐mediated compaction followed by covalent crosslinking on cell compatibility, we performed a fluorescence‐based Live/Dead assay using human dermal fibroblasts (HDFs) as a model cell line (Figure 6A). Viable cells were identified by green fluorescence from intracellular conversion of calcein AM to calcein, whereas dead cells were stained red by ethidium homodimer‐1 after membrane disruption. After 1 day, the cells were predominantly present as individual, mostly spherical cells, consistent with early attachment. Some cells had already begun to spread, indicating initial cell‐matrix interactions with the gelatin‐based substrate. By Day 3, the cell population had increased markedly and formed a denser interconnected layer with elongated fibroblast‐like morphology and local alignment, consistent with robust spreading and the development of cell‐cell contacts on the gelatin‐based matrix [45].

**FIGURE 6:**
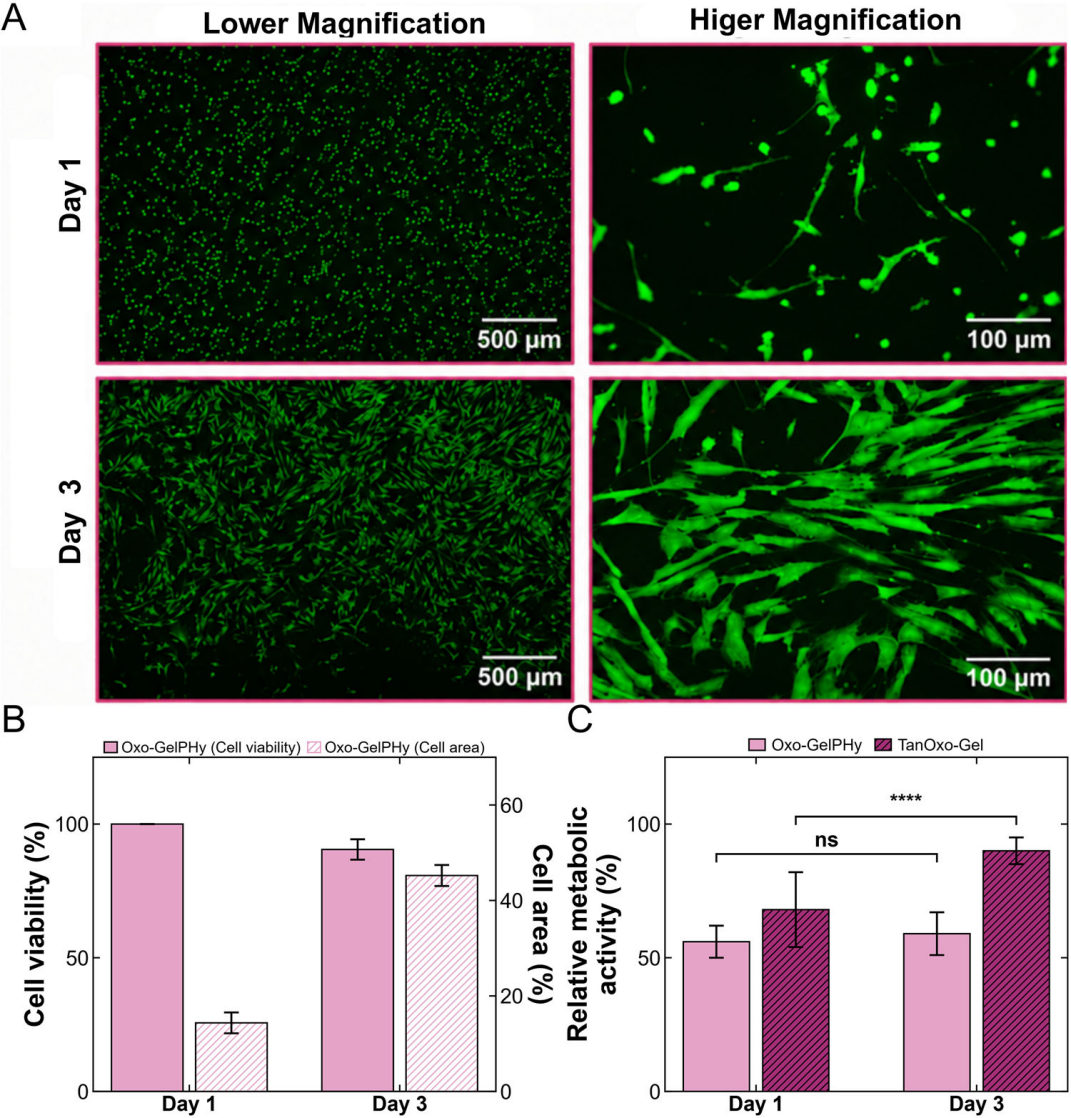
TanOxo‐Gel supports fibroblast viability, spreading, and proliferation. (A) Representative Live/Dead fluorescence micrographs of human dermal fibroblasts (HDFs) cultured on TanOxo‐Gel (1.5 eq) at Days 1 and 3. Images are shown at low and high magnification. (B) Quantification of cell viability (%) (calculated by comparing the number of live cells to the total number of cells using ImageJ analysis of microscopic images) and cell‐covered area (%) at Day 1 and Day 3. (C) Relative metabolic activity of HDFs cultured on TanOxo‐Gel (1.5 eq) and Oxo‐GelPHy (1.5 eq, without tannic acid treatment) at Day 1 and Day 3. Data are shown mean ± standard deviation. Statistical significance was determined via one‐way analysis of variance (ANOVA) followed by Tukey's honestly significant difference (HSD) post hoc test (ns, not significant, ^****^ = *p* < 0.0001).

Analysis of the Live/Dead images showed that HDF viability remained high from Day 1 to Day 3 on TanOxo‐Gel (1.5 eq) (Figure 6B), with only a modest reduction at Day 3 while remaining within a cytocompatible range. In parallel, the cell‐covered area increased markedly between Day 1 and Day 3 (Figure 6B), indicating progressive adhesion, spreading, and surface coverage of the compacted gelatin network. Relative metabolic activity increased significantly from Day 1 to Day 3 on TanOxo‐Gel (1.5 eq), whereas no significant change was observed for the Oxo‐GelPHy control, indicating enhanced time‐dependent metabolic activity on the tannic acid‐compacted network (Figure 6C). These findings are consistent with previous reports showing that tannic acid‐containing gelatin platforms can remain biologically permissive within controlled exposure windows, while still exhibiting concentration‐ and time‐dependent effects [45, 46, 47, 48]. Collectively, these results indicate that TanOxo‐Gel (1.5 eq) maintains high HDF viability while supporting progressive surface coverage and increased metabolic activity over the first 3 days, suggesting that the compaction‐and‐covalent‐crosslinking strategy enhances mechanical performance without compromising early cytocompatibility. Despite acidic processing conditions and the presence of tannic acid, the system remains cytocompatible. This behavior is likely due to sequestration of tannic acid within the compact, covalently stabilized network, which limits the release of free polyphenols and preserves a biologically permissive interface [24]. These findings indicate that mechanical reinforcement can be achieved without compromising early‐stage biological performance [49, 50].

## 3 Conclusions

In summary, we have established a bioinspired strategy that addresses the intrinsic instability of supramolecular gelatin hydrogels by coupling tannic‐acid‐mediated compaction with bis‐oxazoline‐mediated covalent stabilization. By using the acidic microenvironment generated during compaction (pH ≈ 2.7) as an internal trigger, acid‐promoted ring opening of the pre‐embedded bis‐oxazoline enables covalent network formation within the compact gelatin‐rich phase. This integration of a persistent covalent framework with a dynamic, energy‐dissipating supramolecular phase produces a hydrogel with MPa‐level tensile strength, extreme extensibility (≈900%), and substantial hysteretic energy dissipation.

Crucially, covalent stabilization of the compacted state suppresses swelling‐induced structural loss, provides prolonged aqueous dimensional stability, and maintains early cytocompatibility. By translating localized tannic‐acid‐mediated compaction into a macroscopically load‐bearing network, this approach provides a robust route toward water‐stable, protein‐based biomaterials that help bridge the mechanical gap between conventional gelatin hydrogels and native load‐bearing tissues.

## 4 Experimental Section

### 4.1 Materials

Type A gelatin (porcine skin, 300 Bloom), tannic acid (ACS reagent grade, ≥ 99%), 2,2′‐bis(2‐oxazoline) (≥ 98%, herein termed bis‐oxazoline), glycine (≥ 99%), hydrochloric acid (HCl, 37%, ACS reagent grade), deuterium oxide (D_2_O, 99.9 atom % D), and sodium hydroxide (NaOH, ≥ 98%, pellets) were purchased from Sigma–Aldrich and used as received. Deionized water (18.2 MΩ·cm, Milli‐Q) was used throughout.

### 4.2 Preparation of Gelatin Physical Hydrogel (Gel‐PHy)

A 10 wt.% (w/w) gelatin solution was prepared by dissolving gelatin powder in deionized water at 60°C under gentle magnetic stirring until homogeneous. The warm solution was cast into custom square Teflon molds (100 mm × 100 mm, 1.0 mm thickness) and covered with glass plates to prevent evaporation. The molds were incubated at 4°C for 48 h. Circular discs (15 or 20 mm diameter) were punched from the resulting gels for rheological, swelling, and other analyses requiring defined geometries. These hydrogels were used as Gel‐PHy.

### 4.3 Tannic Acid Solution Preparation

Aqueous tannic acid solutions were prepared at 5, 10, 20, 30, and 40 wt.% by dissolving the appropriate mass of tannic acid powder in deionized water under magnetic stirring at room temperature until complete dissolution. The pH of each freshly prepared solution was measured seven times using a calibrated SevenCompact S220 pH meter (*n* = 7).

### 4.4 Effect of Dose‐Dependent Tannic Acid Treatment on Material Properties

Gel‐PHy sheets were immersed in tannic acid solutions (5–40 wt.%) at room temperature. Dimensional changes were monitored every 24 h until equilibrium was reached, typically after 5 days. The top‐down projected surface area (A) was determined from calibrated digital images using ImageJ, and sample thickness (d) was measured using a digital caliper (± 0.01 mm).

Sample volume (V) was calculated according to Equation (1):

(1) V=A×d

The volumetric shrinkage (SV) was calculated relative to the initial volume (V_0_) according to Equation (2):

(2) $$\mathrm{SV}=\frac{\left(V_{0}\;-\;V_{t}\right)}{V_{0}}\;\times \;100\%$$

where Vt is the volume at time t. To isolate the effect of acidity, a dissolution study was performed on Gel‐PHy immersed in HCl solutions with pH values matched to those of the tannic acid solutions (pH 1.0, 2.0, and 3.0). The relative wet mass (M_rel_) was calculated as:

(3) $$M_{\mathrm{rel}}\;=\frac{M_{t}}{M_{0}}\;\;\times 100\%$$

where M_0_ is the initial blotted wet mass. The study was continued for 5 days or until complete dissolution (Mrel = 0%). To distinguish acidification from tannic‐acid‐induced physical compaction in the bis‐oxazoline‐containing system, Oxo‐GelPHy specimens were immersed in an HCl solution adjusted to pH 2.7 without tannic acid. The specimens were incubated for 15 days at 25°C using *n* = 5 independent specimens. Their dimensional response and macroscopic integrity were evaluated using the same volumetric measurement procedure as for the corresponding tannic‐acid‐treated specimens. To quantify the time‐dependent evolution of mechanical properties during tannic acid‐mediated compaction, parallel Gel‐PHy specimens were withdrawn from the tannic acid solutions after 1, 2, 3, and 5 days. Each specimen was gently blotted with lint‐free tissue and surface‐washed by dipping five times for 3 s each in deionized water to remove excess surface tannic acid. The specimens were immediately subjected to oscillatory rheology using the rheological measurement conditions described below.

Based on these optimization studies, 30 wt.% tannic acid was selected for all subsequent hybrid formulations. To further validate this selection, a swelling study before covalent crosslinking was conducted specifically on 30 wt.% tannic acid‐treated samples to evaluate their structural stability and equilibrium mass in deionized water.

### 4.5 Rheological Characterization

Rheological measurements were performed using a TA Instruments DHR‐2 rheometer equipped with a Peltier temperature control system and a smooth 12 mm parallel‐plate geometry at 25°C. Sample thickness was measured using a digital caliper and precisely set as the gap height to ensure proper contact without compression. A constant axial (normal) force of 0.1 N was applied. Amplitude sweeps (0.1%–10% strain at 0.5 Hz) were conducted on all samples to determine the linear viscoelastic region (LVER). For time‐dependent evaluation during tannic acid‐mediated compaction mentioned above, single‐point oscillatory data were acquired at a fixed strain of 0.5% and a frequency of 0.5 Hz. For equilibrated samples, frequency sweeps (0.1–10 Hz) were performed at 0.5% strain within the LVER to record the storage modulus (G′), loss modulus (G″), and tan δ. The selected normal force, measurement gap, and restricted strain range were used to reduce the risk of interfacial slippage, edge fracture, and loss of sample–plate contact. Complete elimination of slippage cannot be assumed for measurements performed using smooth parallel plates.

### 4.6 ATR‐FTIR Characterization of Bis‐Oxazoline‐Crosslinked Gelatin Networks

Freeze‐dried samples were characterized by attenuated total reflection Fourier‐transform infrared spectroscopy (ATR‐FTIR) using a Shimadzu IRTracer‐100 FTIR spectrometer (Shimadzu Corporation, Kyoto, Japan) equipped with an ATR accessory. Spectra were acquired over the range 4000–400 cm^−1^ at a spectral resolution of 4 cm^−1^ by averaging 100 scans per sample. A background spectrum was recorded under the same instrumental conditions before each sample measurement.

Four sample conditions were analyzed: unmodified gelatin; gelatin with 2,2′‐bis(2‐oxazoline) immediately after mixing (*t* = 0); an acid‐mediated bis‐oxazoline hydrolysis control; and the washed Oxo‐GelPHy network. Three replicate samples were analyzed for each condition. All samples were freeze‐dried before ATR‐FTIR analysis.

For the gelatin with bis‐oxazoline immediately after mixing (*t* = 0) control, bis‐oxazoline was added to the gelatin formulation under the initial conditions used for Oxo‐GelPHy preparation. The mixture was freeze‐dried immediately after mixing without completing the crosslinking or washing procedure.

For the acid‐mediated bis‐oxazoline hydrolysis control, the gelatin–bis‐oxazoline mixture was adjusted to pH 2 using HCl and stored at approximately 4°C for 5 days to promote hydrolysis of the oxazoline groups. The resulting sample was subsequently freeze‐dried without undergoing the washing procedure used for Oxo‐GelPHy.

Oxo‐GelPHy was prepared according to the standard crosslinking protocol. Following completion of the reaction, the hydrogel was extensively washed with deionized water to remove unreacted and water‐soluble species and was subsequently freeze‐dried before analysis.

Spectra were exported as percent‐transmittance values and converted to absorbance using A = −log10(T/100), where T represents percent transmittance. Although spectra were acquired over 4000–400 cm^−1^, the exported datasets used for numerical analysis covered 4000–650 cm^−1^. Each absorbance spectrum was normalized independently by division by its mean absorbance within the C─H stretching region at 2890–2970 cm^−1^. The normalized spectra were used for spectral comparison, calculation of difference spectra, local band fitting, and quantitative analysis.

No smoothing, derivative processing, peak sharpening, or global baseline correction was applied. The overview spectra were vertically offset and displayed using an inverted absorbance axis for visualization only. All spectral subtraction, local band fitting, and quantitative analyses were performed using the original, non‐inverted normalized absorbance values.

The difference spectrum was calculated point‐by‐point as the mean C–H‐normalized absorbance spectrum of Oxo‐GelPHy minus the mean C─H‐normalized absorbance spectrum of the acid‐mediated bis‐oxazoline hydrolysis control. Targeted local band fitting was performed independently for each replicate using the non‐smoothed, C─H‐normalized absorbance spectra. Each selected spectral region was fitted using a single pseudo‐Voigt component together with a simultaneously fitted local linear baseline. The carbonyl region was evaluated over 1690–1765 cm^−1^, and the ester‐associated C─O region was evaluated over 1150–1300 cm^−1^. The lower‐wavenumber feature was evaluated separately over 1050–1115 cm^−1^, and its minimum position was determined directly from the group‐mean difference spectrum without peak fitting.

The fitted area of the ester‐associated C─O region was normalized to the corresponding C─H reference‐band area and reported as the C─O/C─H fitted‐area ratio. The fitted‐area ratios are reported as mean ± standard deviation for three replicate samples (*n* = 3). Fit quality was evaluated using the coefficient of determination and root‐mean‐square error.

### 4.7 Macroscopic Thermal‐Stability Comparison

Representative Gel‐PHy, Oxo‐GelPHy, Tan‐GelPHy, and TanOxo‐Gel samples were individually loaded into glass vials and incubated at 60°C for 48 h. The samples were photographed before loading, immediately after loading at 0 h, and after 48 h of incubation. Following incubation, the remaining macroscopically identifiable gel material was removed from the vials and photographed. The experiment was used as a qualitative comparison of macroscopic thermal stability based on the retention or loss of visually identifiable gel material.

### 4.8 Preparation of Bis‐Oxazoline‐Embedded Gelatin Hydrogel (Oxo‐GelPHy)

Bis‐oxazoline was added to the warm 10 wt.% gelatin solution, prepared from the same gelatin precursor solution used for Gel‐PHy, at oxazoline‐to‐carboxylic acid molar equivalents of 0.5–2.0 eq, where [COOH] was estimated from a carboxylic acid content of approximately 0.8 mmol per g dry gelatin:

(4) $$\left(\mathrm{Eq}=\frac{\left[O_{x}\right]}{\left[\mathrm{COOH}\right]}\right)$$

The oxazoline‐to‐carboxylic acid molar ratio (Eq) was thus varied between 0.5 and 2.0. The pH was immediately adjusted to 5.0–5.5 using 0.1 M HCl. The resulting mixture was cast into Teflon molds with thicknesses of 0.5–2.0 mm, depending on the experiment performed, and incubated at 4°C for 48 h to obtain the bis‐oxazoline‐embedded gelatin physical hydrogel (Oxo‐GelPHy). For stability controls, separate specimens were subsequently transferred to 22°C and incubated for an additional 7 days in a humidified chamber.

### 4.9 Optimization of Tannic Acid‐Mediated Compacted Materials and Swelling Studies

Sheets of Gel‐PHy and Oxo‐GelPHy were fully immersed in excess 30 wt.% tannic acid solution (pH 2.7, ≈50 mL per sample) in sealed glass beakers under a nitrogen atmosphere. The beakers were additionally sealed with Parafilm. The protocol consisted of 48 h at 4°C, followed by a further 48 h in the tannic acid solution at 4°C, and then 3 days at 22 ± 1°C, giving a total treatment time of 7 days. This treatment yielded the tannic acid‐treated gelatin physical hydrogel (Tan‐GelPHy) and the tannic acid‐compacted, bis‐oxazoline‐crosslinked gelatin hydrogel (TanOxo‐Gel), respectively.

Tan‐GelPHy specimens used as the tannic‐acid‐only physical‐network control were characterized directly after tannic acid treatment for the volumetric‐stability and rheological analyses. They were not subjected to the complete washing protocol because washing caused pronounced reswelling and dimensional instability in the absence of the bis‐oxazoline‐mediated covalent network.TanOxo‐Gel specimens (2.0 mm initial thickness) were washed with deionized water, which was changed every 12 h for 120 h (eight changes in total). Prior to rheological characterization, the equilibrated TanOxo‐Gel specimens were surface‐washed by dipping five times for 3 s each in deionized water, using the same washing procedure as described previously to remove any surface residue of dried tannic acid. Washed specimens were then transferred to Petri dishes containing 50 mL deionized water at 25°C. Wet mass (m_wet_) was recorded after gentle blotting with lint‐free tissue until no visible surface water remained at 1, 2, 3, and 5 days (*n* = 7). The swelling ratio was calculated as:

(5) $$Q\left(t\right)=\frac{m_{wet}}{m_{initial}}$$

where m_initial_ is the mass of the as‐prepared hydrogel immediately before tannic acid treatment. For the long‐term aqueous stability study, hydrogel volume was determined at D0 and after 1, 3, 7, 14, and 21 days of immersion in deionized water at 25°C. Volume was determined from calibrated top‐down images and sample‐thickness measurements as described above. Formulations that lost macroscopic integrity were recorded as disintegrated and were not assigned a quantitative volumetric value at the corresponding time point. The 0.50 and 0.75 eq Oxo‐GelPHy formulations were recorded as disintegrated at Day 21.

### 4.10 Mechanical Tensile Testing

Hydrogel sheets were cast into rectangular Teflon molds (200 × 10 × 0.5 mm). The mold assembly was covered with a glass plate and subjected to a static load (5 kg) during gelation to ensure uniform thickness and surface flatness. After washing and equilibration as described above, dumbbell‐shaped specimens (*n* = 7 per group) were punched from TanOxo‐Gel sheets containing 1.25, 1.5, and 1.75 eq bis‐oxazoline. For the mechanical control, equilibrated Oxo‐GelPHy specimens prepared without tannic acid‐mediated compaction were used. Tests were performed at room temperature (25°C, 70%–90% relative humidity) using a mechanical tester equipped with a 50 N load cell at a strain rate of 50 mm min^−1^. Young's modulus, ultimate tensile strength, and elongation at break were recorded.

Cyclic loading was performed to assess hysteresis and energy dissipation. To avoid load‐history effects, separate specimens (*n* = 3) were subjected to five consecutive loading‐unloading cycles at fixed peak strains corresponding to 25%, 50%, and 75% of the formulation‐specific ultimate elongation at break (εb). The dissipated energy density was determined from the area enclosed by the loading–unloading hysteresis loop for each cycle in TanOxo‐Gel (1.25, 1.5, and 1.75 eq bis‐oxazoline) and the corresponding Oxo‐GelPHy controls. The cumulative dissipated energy density was calculated as the sum of the individual cycle contributions [31].

### 4.11 In Vitro Cell Culture and Cytocompatibility

Human dermal fibroblasts (HDFs) were expanded in T‐150 culture flasks using Dulbecco’s Modified Eagle Medium (low glucose, Gibco) supplemented with 10% fetal bovine serum (Gibco) and 1% antibiotic–antimycotic. Cultures were maintained in a humidified atmosphere at 37°C and 5% CO_2_, with the medium replenished every 48 h. Upon reaching ≈80% confluency, cells were harvested using TrypLE for detachment. HDFs between passages 8 and 10 were used for all biological evaluations.

To assess cytocompatibility, HDFs were seeded on TanOxo‐Gel containing 1.5 eq bis‐oxazoline. Prior to cell seeding, hydrogel specimens were sterilized by sequential exposure to 70% ethanol and ultraviolet irradiation (30 min per side), followed by triple washing with sterile phosphate‐buffered saline (PBS) and complete culture medium. Specimens were equilibrated overnight in culture medium at 37°C to ensure physiological stability. Sterilized constructs were transferred to 24‐well plates, and HDFs were seeded on the hydrogels at a density of 30 000 cells per construct.

Live/Dead staining (Live/Dead Viability/Cytotoxicity Kit for Mammalian Cells, ThermoFisher) was performed after 24 h (Day 1) and 72 h (Day 3). At each time point, the medium was removed, and the samples were washed with PBS, followed by incubation with Live/Dead staining solution prepared in culture medium (2 µL mL^−1^ Calcein AM and 4 µL mL^−1^ Ethidium homodimer‐1) for 1 h at 37°C protected from light. After incubation, the samples were washed again with PBS and imaged using a Nikon Eclipse Ts2 fluorescence microscope with 3× and 15× objectives. For quantification of cell coverage at Day 1 and 3, images acquired with the 15× objective were analyzed using ImageJ (National Institutes of Health, USA) with a threshold‐based segmentation approach to identify cell‐positive regions, and cell coverage was expressed as the percentage of the imaged surface area occupied by cells relative to the total imaged area. Measurements were performed in triplicate per condition and are reported as mean ± SD.

The metabolic activity of the cells was determined using the PrestoBlue assay (ThermoFisher). Briefly, HDFs were cultured on both TanOxo‐Gel (1.5 eq bis‐oxazoline) and Oxo‐GelPHy (1.5 eq bis‐oxazoline, non‐tanned) and were analyzed at Day 1 and 3. At the designated time points, 500 µL of a 10% (v/v) PrestoBlue solution in complete medium was added to each well and incubated for 6 h at 37°C. The supernatant was transferred to a 96‐well plate, and fluorescence intensity excitation 555 nm / emission 595 nm was recorded using a microplate reader. Results were normalized to a control consisting of 30 000 cells seeded on tissue culture plastic at the corresponding time point.

### 4.12 Statistical Analysis

All experiments were performed at least in triplicate. Statistical analyses were performed using Python. Depending on the dataset, differences between groups were assessed using one‐way or two‐way analysis of variance. A p‐value < 0.05 was considered statistically significant. Significance levels were defined as follows: ^****^ *p* < 0.0001, ^***^ *p* < 0.001, ^**^ *p* < 0.01, and ^*^ *p* < 0.05.

## Conflicts of Interest

The authors declare no conflicts of interest.

## Declaration of Generative AI and AI‐Assisted Technologies in the Manuscript Preparation Process

During the preparation of this work, the authors used the ChatGPT language model in order to polish the English presentation. After using this service, the authors reviewed and edited the content as needed and take full responsibility for the content of the published article.

## Supporting information




**Supporting File 1**: smll75441‐sup‐0001‐SuppMat.pdf.

**Supporting File 2**: smll75441‐sup‐0002‐VideoS1‐S9.zip.

## Data Availability

The data supporting the findings of this study are available from the corresponding author upon reasonable request.
