# Supplementary material for: Ultra‐Tough Hybrid Supramolecular Gelatin Hydrogels
Source: Small. 2026 Sep 2;22(61):e75441. doi: 10.1002/smll.75441 (PMC13725415; doi:10.1002/smll.75441)
Supplement: Supplementary file 1 [file SMLL-22-e75441-s002.pdf]

Supporting Information

Ultra-Tough Hybrid Supramolecular Gelatin Hydrogels

Patrick Shakari,[†1] Christos Leliopoulos,[†2] Hamidreza Mokhtari,[2] Rohith Pavan Parvathaneni,[2] Afroditi Kontse, Jöns Hilborn,[1] and Oommen P. Varghese[2*]*

[1]Program of Macromolecular Chemistry, Department of Chemistry - Ångström Laboratory, Uppsala University, Box 538, SE-751 21 Uppsala, Sweden

[2]Translational Chemical Biology Group, Science for Life Laboratory, Division of Macromolecular Chemistry, Department of Chemistry-Ångstrom Laboratory, Uppsala University, Uppsala SE75121, Sweden

Table of Contents

Figure S1. Tannic acid-induced compaction and viscoelastic evolution of the physical gelatin network.
Figure S2. Acidic control studies for bis-oxazoline crosslinking.
Figure S3. Rheological evolution of Oxo-GelPHy and TanOxo-Gel as a function of bis-oxazoline equivalent and incubation time.
Figure S4. pH-matched acidity control for Oxo-GelPHy.
Figure S5. ATR-FTIR characterization of bis-oxazoline-crosslinked gelatin networks.
Figure S6. Macroscopic thermal-stability comparison of Gel-PHy, Oxo-GelPHy, Tan-GelPHy, and TanOxo-Gel.
Figure S7. Tensile stress–strain behavior of Oxo-GelPHy and TanOxo-Gel.
Figure S8. Cyclic tensile behavior and dissipated-energy analysis of Oxo-GelPHy and TanOxo-Gel.

Figure S1. Tannic acid concentration-dependent compaction and viscoelastic evolution of Gel-PHy.

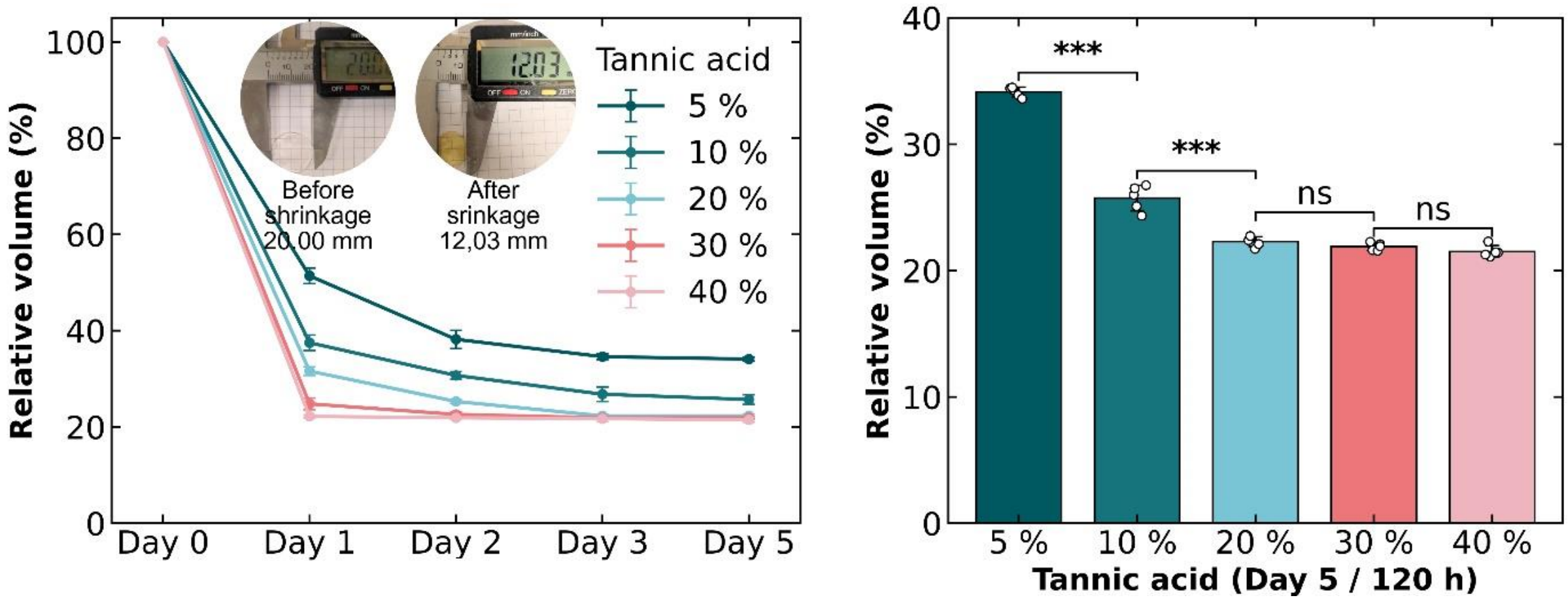

Figure S1A. (A) Relative volume (%) of Gel-PHy during immersion in 5, 10, 20, 30, and 40 wt% tannic acid over 5 days. All samples contracted rapidly within the first 24 h, followed by a slower approach to a plateau. Higher tannic acid concentrations (20-40 wt%) induced greater shrinkage than 5-10 wt%. Insets show representative images before and after shrinkage. **(B)** Relative volume (%) at Day 5 (120 h) for the corresponding tannic acid concentrations. The 5 and 10 wt% groups retained significantly higher relative volume than the 20-40 wt% groups, whereas no significant differences were observed among the 20, 30, and 40 wt% groups. Data are presented as mean ± SD. Statistical significance is indicated in the figure and was determined by one-way ANOVA with Tukey's HSD post hoc test (ns, not significant; ***$p < 0.001$).

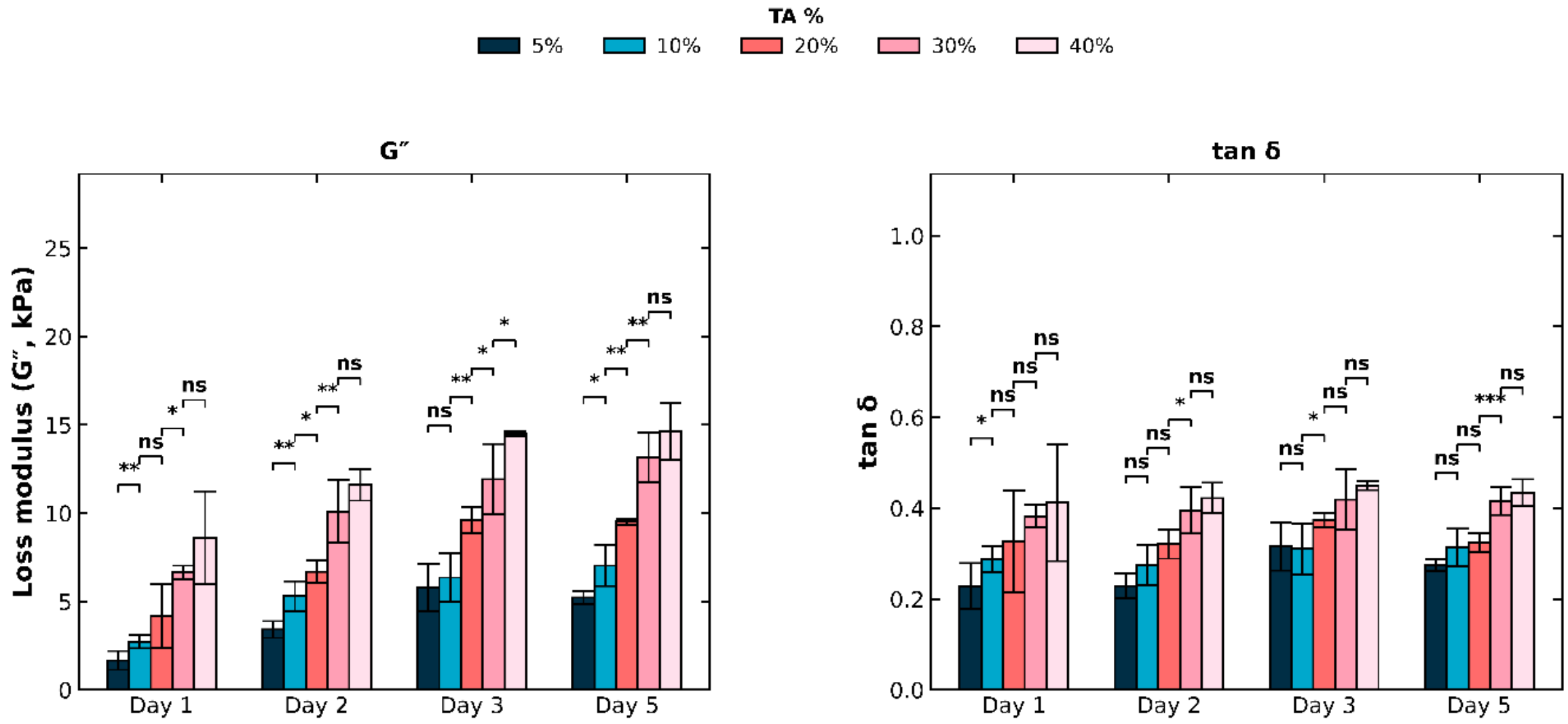

Figure S1B. Loss modulus (G″, left) and loss factor (tan δ**,** right) of tannic acid-treated gelatin hydrogels prepared with 5-40 wt% tannic acid and measured after 1, 2, 3, and 5 days of incubation. Both G″ and tan δ increased with tannic acid concentration at all time points, indicating progressively greater viscous dissipation and a more dissipative viscoelastic response in the more strongly compacted networks. The highest values were consistently observed for the 30-40 wt% tannic acid formulations, whereas the 5-10 wt% formulations remained less dissipative. Data are presented as mean ± SD. Statistical significance was determined **by** two-way ANOVA with Tukey's HSD post hoc test (ns, not significant; $p < 0.05$, $p < 0.01$**,** $p < 0.001$).

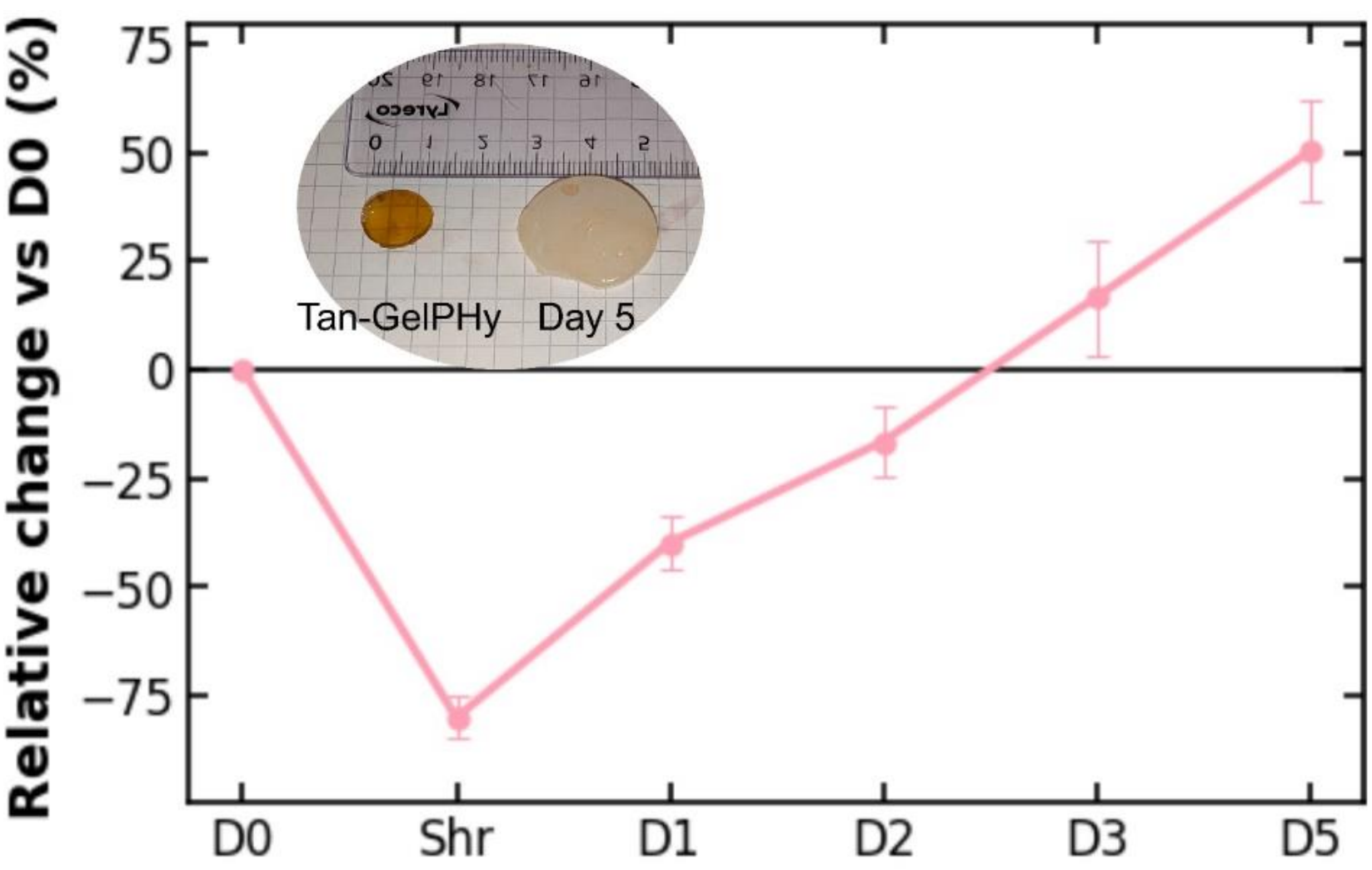

*Figure S1C. Purely physical tannic acid-gelatin hydrogels show rapid compaction but limited volumetric stability in water***.** *Relative volumetric change of Tan-GelPHy relative to D0 during tannic acid-induced compaction (Shr) and subsequent immersion in water (D1–D5). The hydrogel contracted rapidly upon tannic acid treatment but progressively re-swelled during*

water incubation and exceeded its initial volume by D3–D5. The initially compact brown hydrogel also became markedly paler and opaque after 5 days in water. The inset shows representative photographs of Tan-GelPHy immediately after tannic acid-induced compaction and after 5 days of immersion in water. Data are presented as mean ± SD.

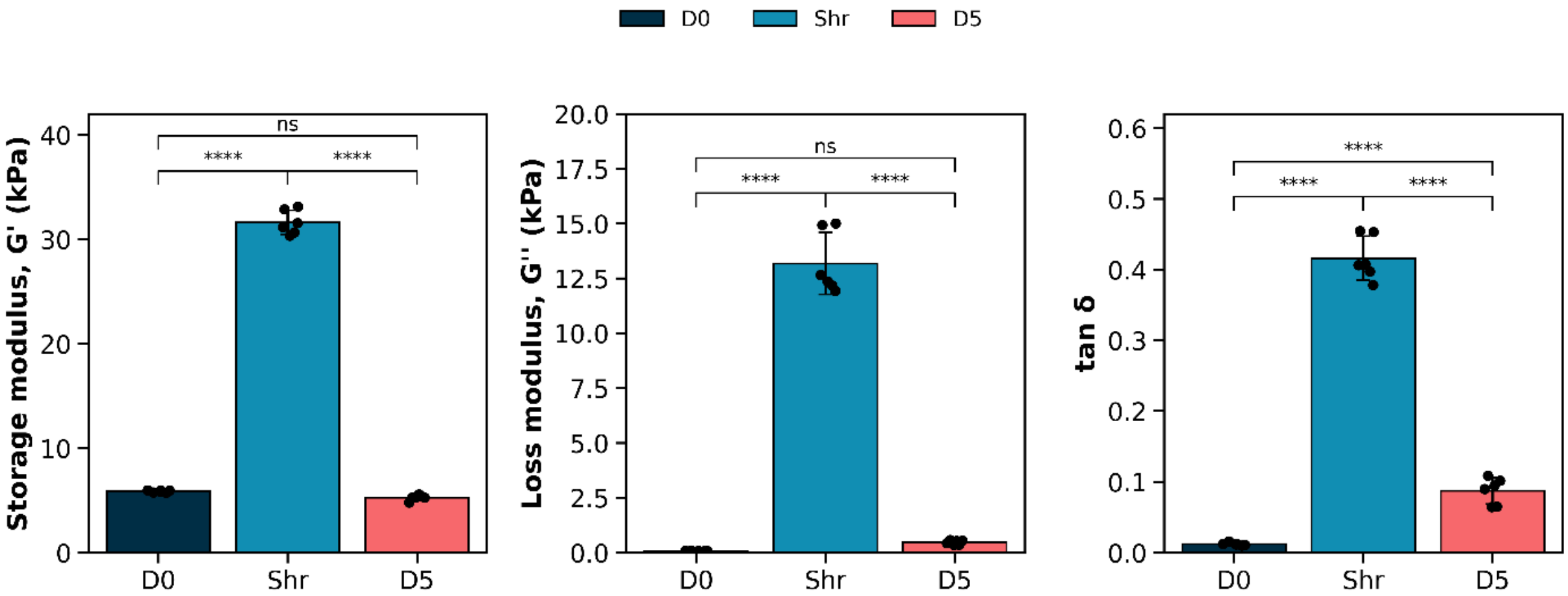

Figure S1D.Tannic acid treatment transiently increases viscoelasticity during compaction, followed by partial recovery after equilibration in water**.** Storage modulus (G′), loss modulus (G″), and loss factor (tan δ) of pristine gelatin hydrogels at the initial state (D0), after tannic acid-induced compaction (Shr), and after subsequent equilibration in water (D5). Tannic acid treatment produced a pronounced increase in both G′ and G″**,** together with a marked rise in tan δ, indicating formation of a highly dissipative compacted state. After transfer to water, G′**,** G″, and tan δ decreased substantially and approached values closer to the initial state, consistent with partial structural relaxation of the purely physical network. Data are presented as mean ± SD. Statistical significance was determined by one-way ANOVA with Tukey's multiple-comparisons test (ns, not significant; ****$p < 0.0001$).

Figure S2. Acidic treatment alone induces swelling and destabilization of Gel-PHy**.**

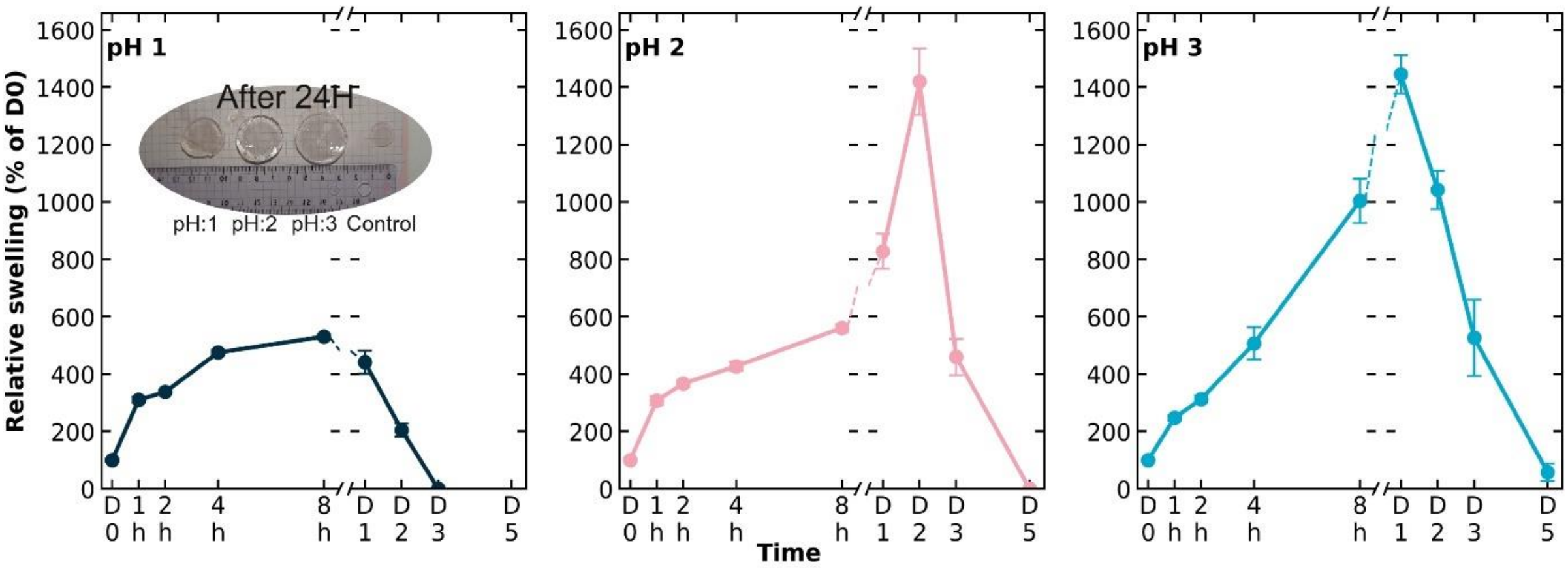

Figure S2A. Acidic treatment alone induces pronounced swelling of pristine gelatin hydrogels, with the strongest response at pH 2-3.**(**A) Relative swelling of pristine gelatin hydrogels at pH 1 as a function of time, reported as % of the initial state (D0). The inset shows representative photographs after 24 h at pH 1-3 together with the untreated control.(B) Relative swelling of pristine gelatin hydrogels at pH 2 as a function of time. (C) Relative swelling of pristine gelatin hydrogels at pH 3 as a function of time. All conditions showed rapid swelling during the first 8 h, followed by a delayed maximum and a subsequent decrease at later time points. The swelling response was lowest at pH 1, whereas pH 2 and pH 3 produced markedly higher transient swelling. Data are presented as mean ± SD.

Figure S3. Viscoelastic properties of Oxo-GelPHy and TanOxo-Gel as a function of bis-oxazoline equivalent and incubation time.

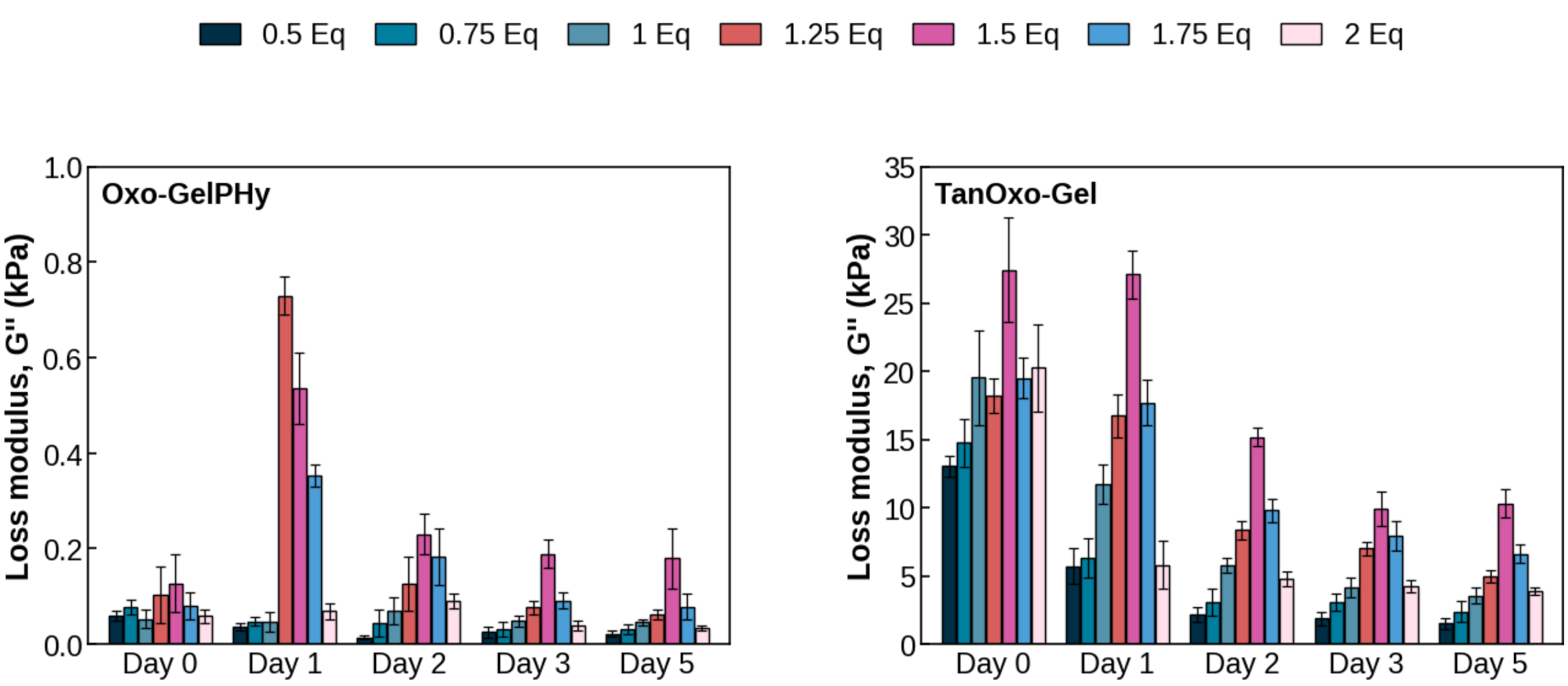

Figure S3A. Tannic acid-mediated compaction preserves a substantially higher loss modulus after covalent crosslinking than covalent crosslinking alone. Loss modulus (G") of Oxo-

GelPHy (left) and TanOxo-Gel (right) as a function of bis-oxazoline loading (0.5-2.0 eq) and immersion time (Day 0-Day 5). Oxo-GelPHy remained in a low G" regime throughout the time course, whereas TanOxo-Gel exhibited markedly higher G" values across the screened formulations, indicating retention of a stronger dissipative viscoelastic component after tannic acid-mediated compaction and covalent crosslinking. Among the compacted formulations, the 1.5 eq sample showed the highest G" at early time points and retained comparatively high values after equilibration. Data are presented as mean ± SD.

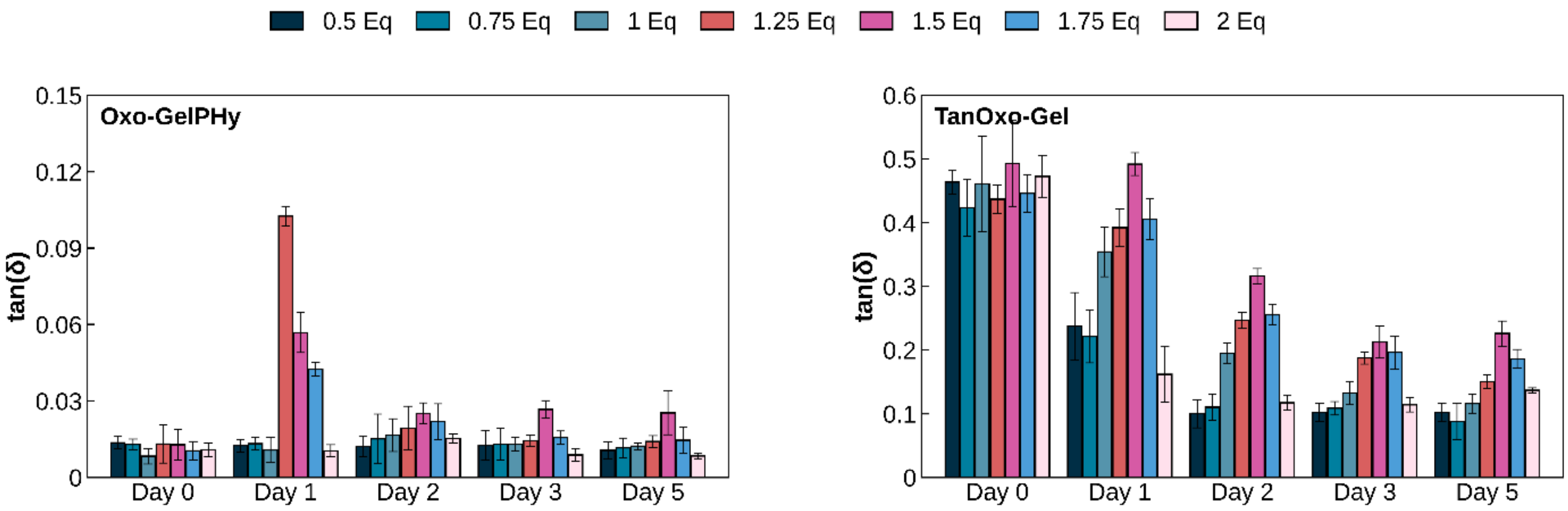

Figure S3B. Tannic acid-mediated compaction preserves a substantially higher viscoelastic contribution than covalent crosslinking alone. Tan δ of Oxo-GelPHy (left) and TanOxo-Gel (right) as a function of bis-oxazoline loading (0.5-2.0 eq) and immersion time **(**Day 0-Day 5**).** The lower tan δ range of Oxo-GelPHy indicates a less dissipative response after covalent crosslinking in the absence of tannic acid-mediated compaction. In contrast, TanOxo-Gel exhibited substantially higher tan δ values across the screened formulations, consistent with retention of a supramolecular dissipative component after compaction and covalent crosslinking. Among the compacted formulations, the 1.5 eq sample retained the highest tan δ after equilibration, indicating the most favorable balance between retained dissipation and network stabilization. Data are presented as mean ± SD.

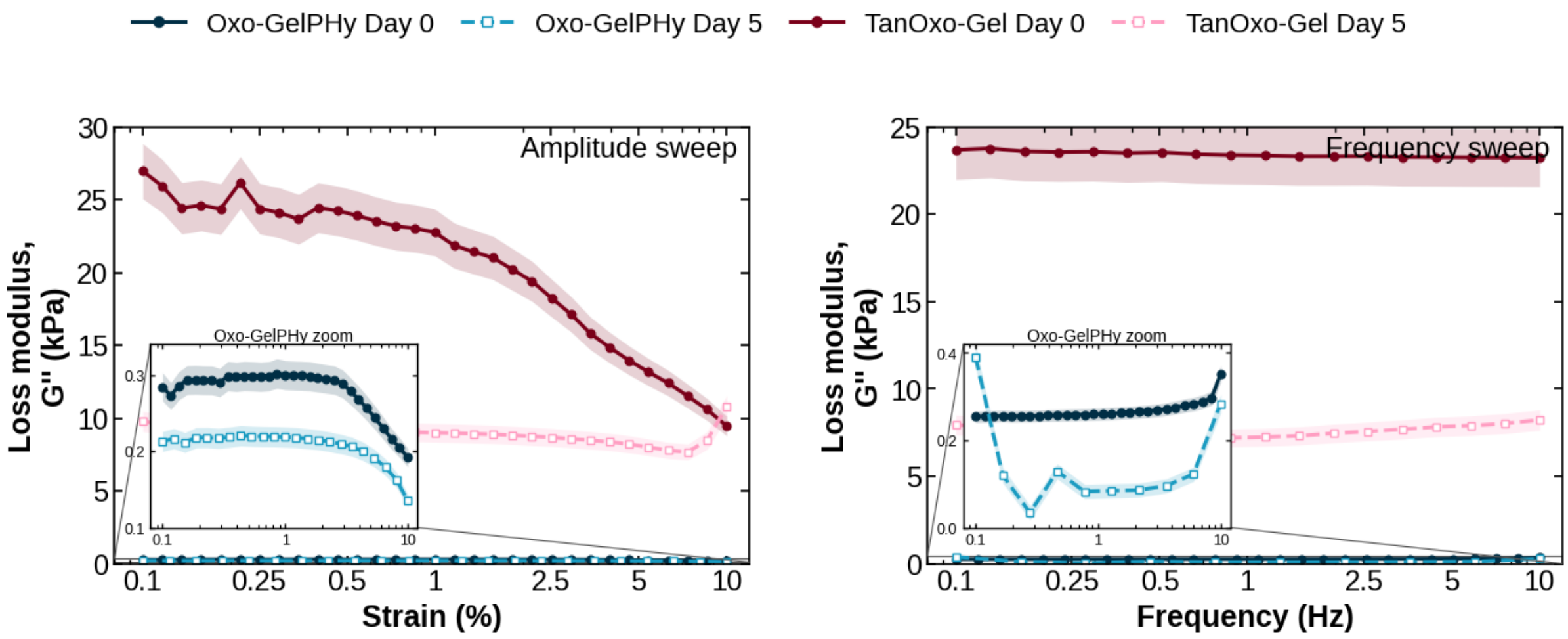

Figure S3C. Rheological loss response of Oxo-GelPHy and TanOxo-Gel at Day 0 and Day 5. Oscillatory amplitude sweeps showing the loss modulus (G″) as a function of strain from 0.1% to 10% at 0.5 Hz, together with frequency sweeps showing G″ from 0.1 to 10 Hz at a fixed strain of 0.5%. Measurements were performed at 25 °C for Oxo-GelPHy and TanOxo-Gel containing 1.50 eq bis-oxazoline. Solid lines denote Day 0 and dashed lines denote Day 5. Lines represent mean values and shaded regions indicate standard deviation (n = 7).

Figure S4. pH-matched acidity control for Oxo-GelPHy.

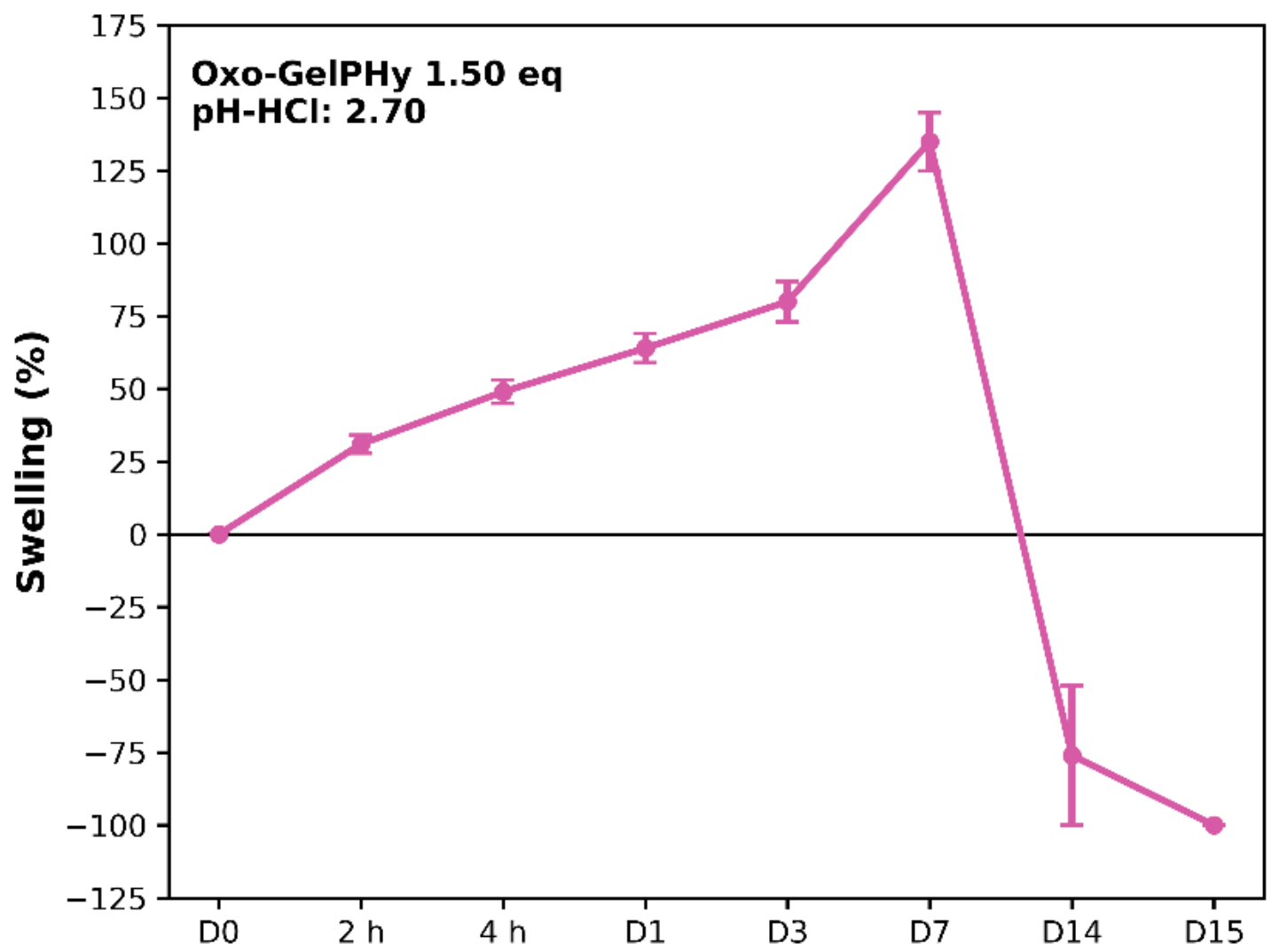

Figure S4. pH-matched acidity control for Oxo-GelPHy. Time-dependent swelling of Oxo-GelPHy prepared with 1.50 eq bis-oxazoline during immersion in an HCl solution adjusted to pH 2.7 without tannic acid, referenced to the initial state (D0). The hydrogel exhibited progressive swelling during the first 7 days, increasing from approximately 31% after 2 h to approximately 135% at Day 7. This was followed by pronounced loss of dimensional and

macroscopic stability, with the measured value decreasing to approximately −76% at Day 14 and −100% at Day 15. Data are presented as mean ± SD (n = 5).

Figure S5. Control-resolved ATR-FTIR analysis of bis-oxazoline-mediated chemical network formation in gelatin.

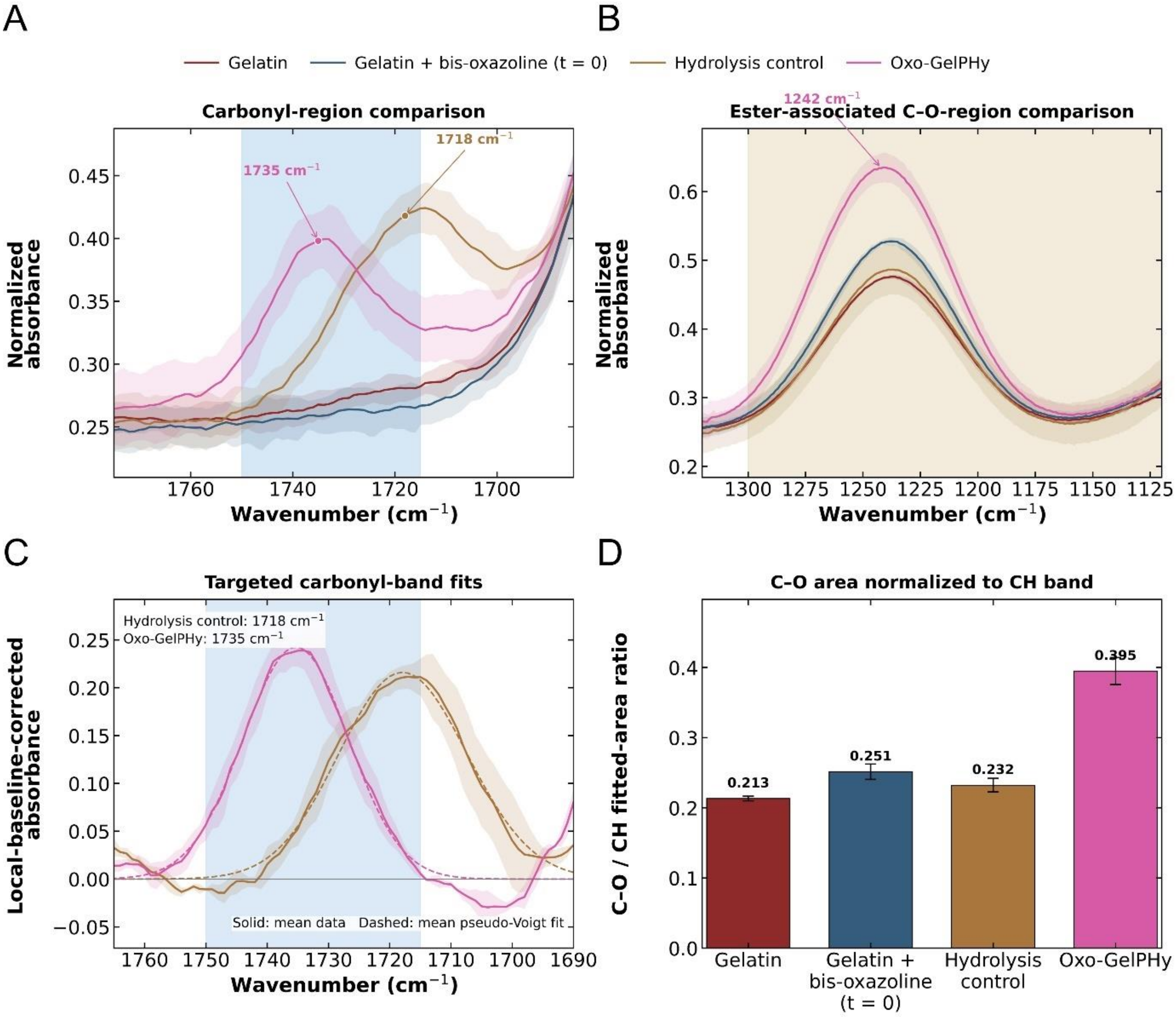

Figure S5. Detailed ATR-FTIR analysis of bis-oxazoline-mediated chemical network formation. (A) Carbonyl-region comparison showing band maxima near 1735 cm^{-1} for Oxo-GelPHy and 1718 cm^{-1} for the hydrolysis control. (B) Ester-associated C–O-region comparison showing an enhanced contribution in Oxo-GelPHy near 1242 cm^{-1}. (C) Targeted local carbonyl-band fits of the hydrolysis control and Oxo-GelPHy. Solid lines represent the mean baseline-corrected spectra, and dashed lines represent the corresponding pseudo-Voigt fits. (D) Ester-associated C–O/C–H fitted-area ratio for the four analyzed conditions. Data are presented as mean ± SD (n = 3).

Figure S6. Macroscopic thermal-stability comparison of Gel-PHy, Oxo-GelPHy, Tan-GelPHy, and TanOxo-Gel.

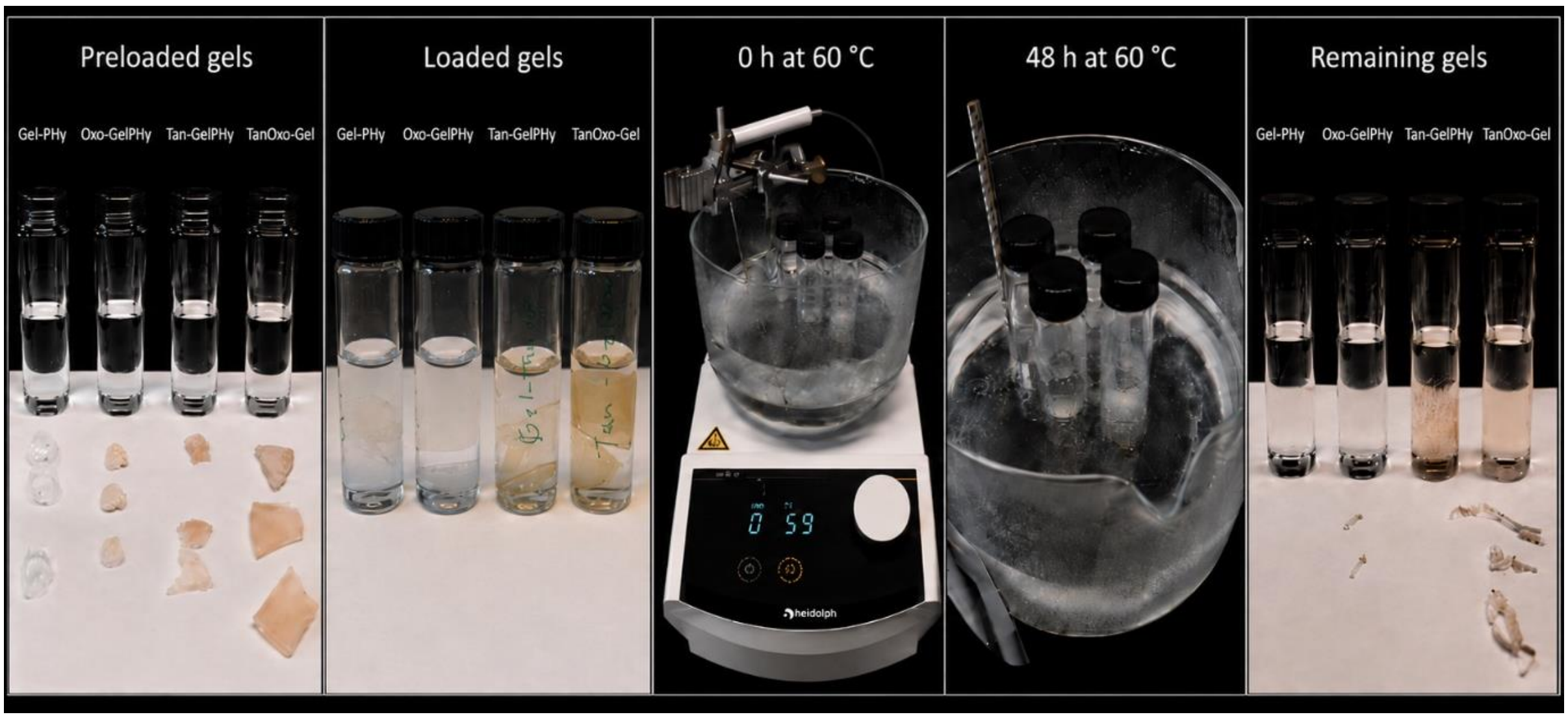

Figure S6. Macroscopic thermal-stability comparison of Gel-PHy, Oxo-GelPHy, Tan-GelPHy, and TanOxo-Gel. Representative photographs show the gel materials before loading, after loading into glass vials, at the beginning of the thermal treatment, after incubation at 60 °C for 48 h, and the remaining gel material recovered after incubation. Gel-PHy and Tan-GelPHy showed pronounced loss of macroscopic integrity, whereas Oxo-GelPHy and TanOxo-Gel retained visually identifiable gel material. This experiment provides complementary evidence of thermally persistent network stabilization and is not interpreted as independent structural proof of a specific covalent linkage.

Figure S7. Tensile stress-strain behavior of Oxo-GelPHy and TanOxo-Gel.

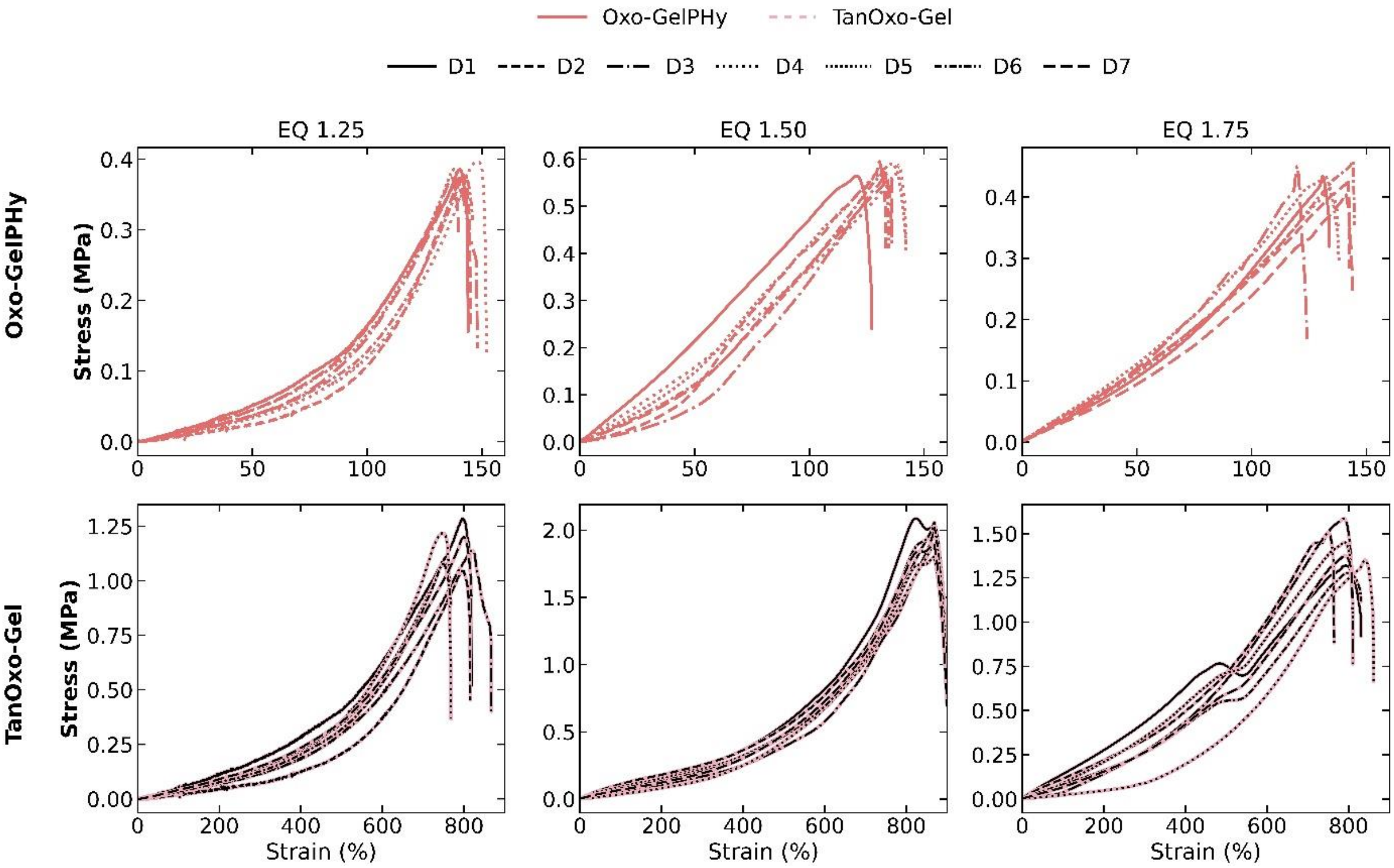

Figure S7A. Tannic acid-mediated compaction markedly enhances large-strain tensile performance across the screened bis-oxazoline loadings**.** Representative engineering stress-strain curves of Oxo-GelPHy (top row) and TanOxo-Gel (bottom row) after immersion for D1-D7, shown for 1.25 eq**,** 1.5 eq**,** and 1.75 eq bis-oxazoline. Line style denotes immersion day, as indicated in the legend. Across all three formulations, TanOxo-Gel exhibited substantially higher stress levels and much greater extensibility than the corresponding Oxo-GelPHy controls. The largest tensile response was observed for TanOxo-Gel at 1.5 eq, whereas Oxo-GelPHy remained limited to lower stress and earlier failure throughout the time course. Overall, the figure shows that tannic acid-mediated compaction, combined with covalent crosslinking, produces a mechanically stronger and more extensible network than covalent crosslinking alone.

Videos S1-S3. Tensile deformation and stress-induced whitening of TanOxo-Gel. Real-time video recordings of monotonic tensile testing of TanOxo-Gel prepared with 1.25, 1.5, and 1.75 Eq bis-oxazoline. The videos show the high extensibility of the hydrogels and the appearance of localized whitening in the gauge region immediately before fracture.

Videos SV1-S6 Monotonic tensile deformation

SV1. Monotonic tensile deformation of TanOxo-Gel (1.25 eq). Real-time video recording of monotonic tensile testing of TanOxo-Gel prepared with 1.25 eq bis-oxazoline.

SV2. Monotonic tensile deformation of TanOxo-Gel (1.5 eq). Real-time video recording of monotonic tensile testing of TanOxo-Gel prepared with 1.5 eq bis-oxazoline.

SV3. Monotonic tensile deformation of TanOxo-Gel (1.75 eq). Real-time video recording of monotonic tensile testing of TanOxo-Gel prepared with 1.75 eq bis-oxazoline.

SV4. Monotonic tensile deformation of Oxo-GelPHy (1.25 eq). Real-time video recording of monotonic tensile testing of Oxo-GelPHy prepared with 1.25 eq bis-oxazoline.

SV5. Monotonic tensile deformation of Oxo-GelPHy (1.5 eq). Real-time video recording of monotonic tensile testing of Oxo-GelPHy prepared with 1.5 eq bis-oxazoline.

SV6. Monotonic tensile deformation of Oxo-GelPHy (1.75 eq). Real-time video recording of monotonic tensile testing of Oxo-GelPHy prepared with 1.75 eq bis-oxazoline.

Figure S8. Cyclic tensile behavior and energy dissipation of Oxo-GelPHy and TanOxo-Gel.

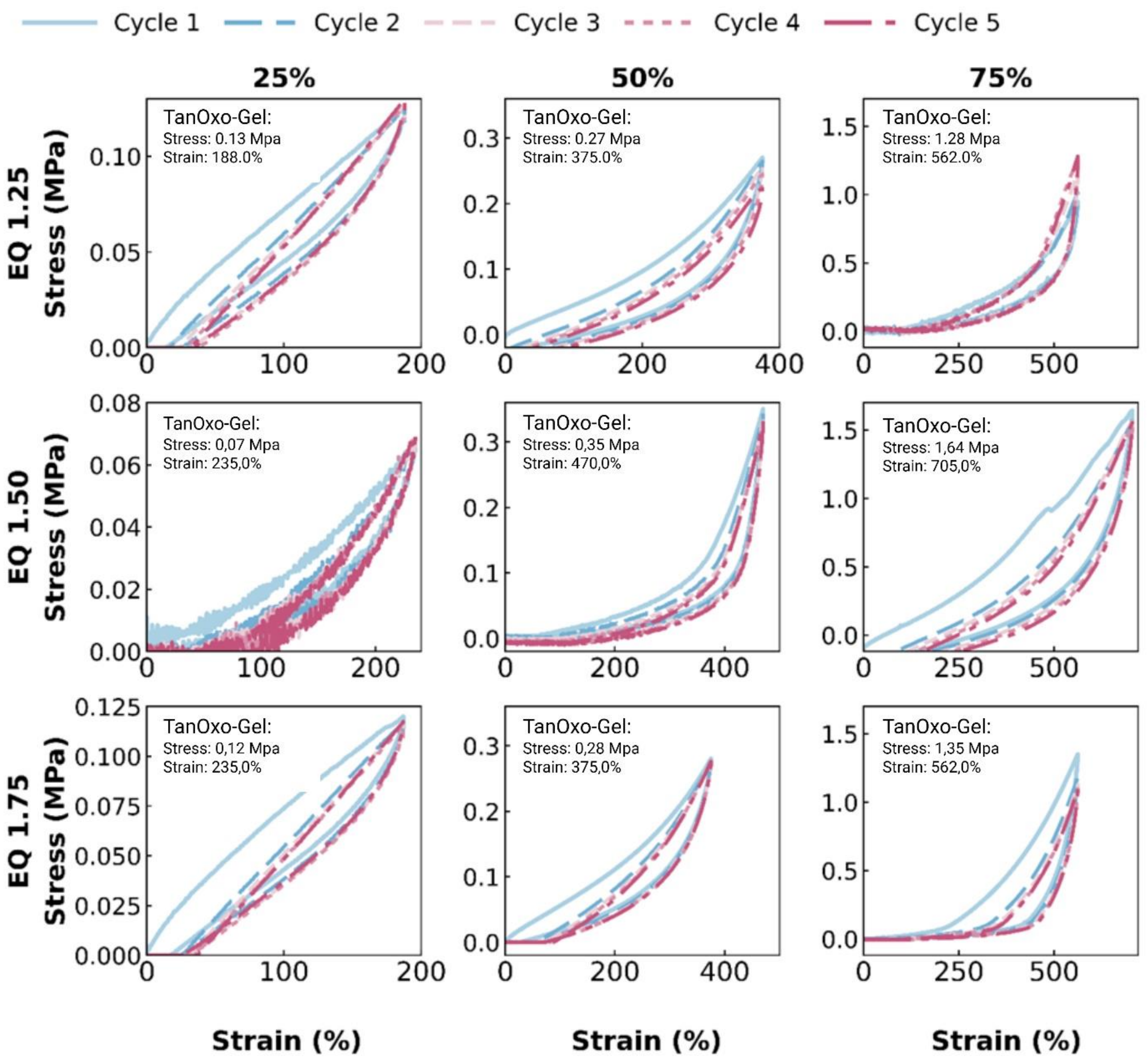

Figure S8A. Cyclic loading-unloading stress-strain behavior of TanOxo-Gel at different bis-oxazoline equivalents and strain levels. Engineering stress–strain curves recorded over five consecutive loading–unloading cycles for TanOxo-Gel prepared with 1.25, 1.5, and 1.75 eq bis-oxazoline (rows) and tested at target strains corresponding to 25%, 50%, and 75% of the formulation-specific ultimate elongation at break (columns).In all groups, the first cycle shows the highest stress response, followed by lower and more closely overlapping subsequent cycles with clear hysteresis. Increasing strain level is associated with higher peak stress in all formulations, with the highest stress response observed for the 1.5 Eq group at 75% strain. The corresponding strength and strain values are indicated in each panel.

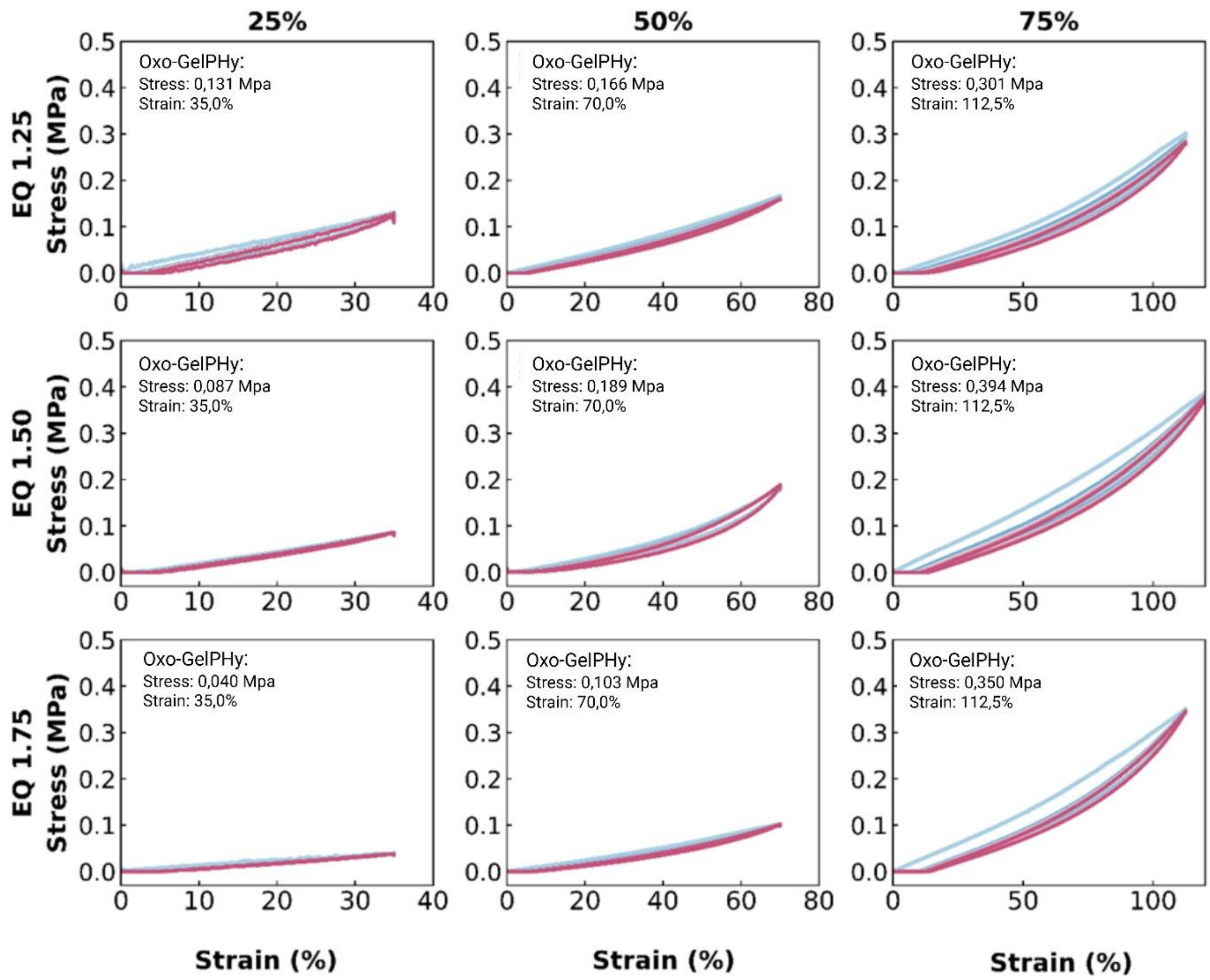

Figure S8B. Cyclic tensile loading reveals strain- and formulation-dependent hysteresis in Oxo-GelPHy . Representative cyclic engineering stress-strain curves of Oxo-GelPHy at 1.25 eq, 1.5 eq, and 1.75 eq bis-oxazoline under sequential loading-unloading to fixed peak strains corresponding to 25%, 50%, and 75% of the formulation-specific ultimate elongation at break. Each panel shows the repeated loading response at the indicated target strain, and the inset values report the corresponding peak stress and strain. At 25% and 50% target strain, the cyclic response decreased with increasing bis-oxazoline loading, whereas at 75% target strain the highest stress was observed for the 1.5 eq formulation. Overall, the figure shows that the magnitude of hysteresis and stress response depends on both deformation level and crosslinker loading, with the most pronounced large-strain response observed at 1.5 eq.

Videos S7-S9. Cyclic loading-unloading of TanOxo-Gel.

SV7. Cyclic tensile deformation of TanOxo-Gel (1.5 eq) at 25% strain. Real-time video recording of cyclic loading-unloading of TanOxo-Gel prepared with 1.5 eq bis-oxazoline over five cycles at a target strain of 25%.

SV8. Cyclic tensile deformation of TanOxo-Gel (1.5 eq) at 50% strain. Real-time video recording of cyclic loading-unloading of TanOxo-Gel prepared with 1.5 eq bis-oxazoline over five cycles at a target strain of 50%.

SV9. Cyclic tensile deformation of TanOxo-Gel (1.5 eq) at 75% strain. Real-time video recording of cyclic loading-unloading of TanOxo-Gel prepared with 1.5 eq bis-oxazoline over five cycles at a target strain of 75%.

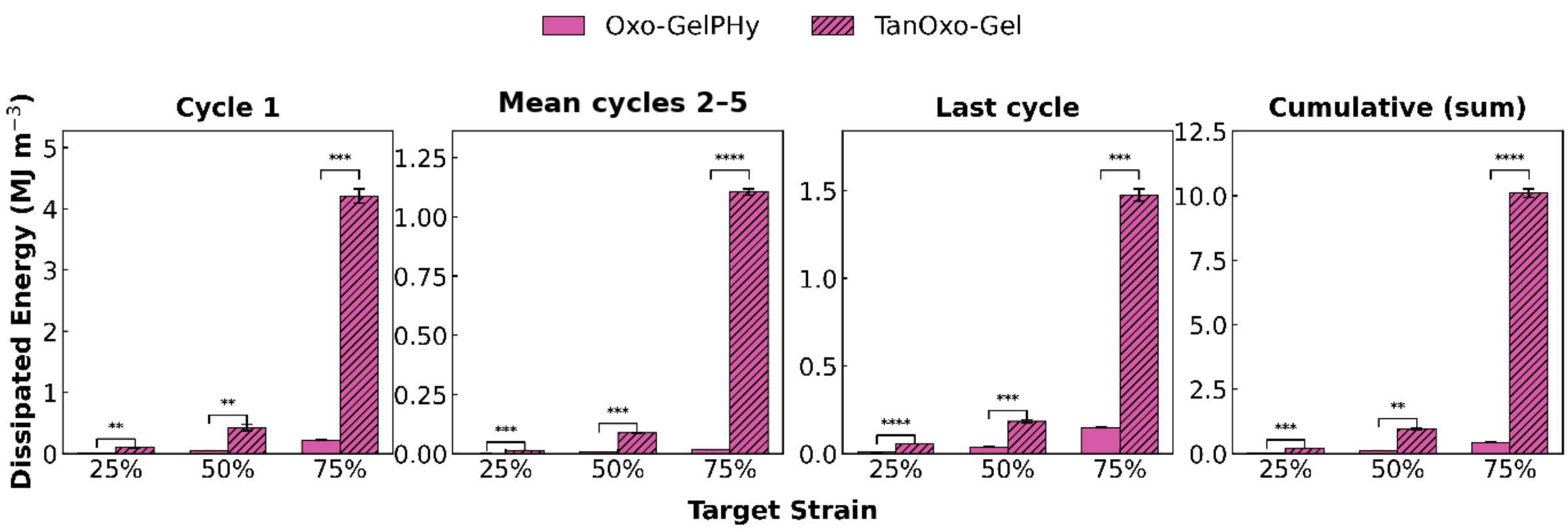

Figure S8C. Dissipated energy density of Oxo-GelPHy and TanOxo-Gel during sequential cyclic tensile loading to target strains corresponding to 25%, 50%, and 75% of the formulation-specific ultimate elongation at break. Panels show the dissipated energy density for Cycle 1, the mean of Cycles 2–5, Cycle 5, and the cumulative sum over the complete five-cycle loading protocol. At all target strains, TanOxo-Gel dissipated more energy than the corresponding Oxo-GelPHy control, with the largest difference observed at the 75% target strain. The cumulative dissipated energy was particularly high for TanOxo-Gel at the 75% target strain, indicating sustained energy dissipation during repeated loading. Data are presented as mean ± SD. Statistical significance was determined by two-way ANOVA (ns, not significant; $p < 0.05$, $p < 0.01$, $p < 0.001$, $p < 0.0001$).
